# KS18, a Mcl-1 inhibitor, improves the effectiveness of bortezomib and overcomes resistance in refractory multiple myeloma by triggering intrinsic apoptosis

**DOI:** 10.3389/fphar.2024.1436786

**Published:** 2024-10-01

**Authors:** Omar S. Al-Odat, Weam Othman Elbezanti, Krishne Gowda, Sandeep K. Srivastava, Shantu G. Amin, Subash C. Jonnalagadda, Tulin Budak-Alpdogan, Manoj K. Pandey

**Affiliations:** ^1^ Department of Biomedical Sciences, Cooper Medical School of Rowan University, Camden, NJ, United States; ^2^ Department of Chemistry and Biochemistry, College of Science and Mathematics, Rowan University, Glassboro, NJ, United States; ^3^ Department of Surgery, Cooper University Health Care, Camden, NJ, United States; ^4^ Department of Pharmacology, Penn State Hershey Cancer Institute, Penn State College of Medicine, Hershey, PA, United States; ^5^ Department of Biosciences, Manipal University Jaipur, Jaipur, Rajasthan, India; ^6^ Department of Hematology, Cooper University Health Care, Camden, NJ, United States

**Keywords:** multiple myeloma (MM), Bcl-2, Mcl-1, bortezomib, venetoclax, refractory, drug resistance

## Abstract

Despite a record number of clinical studies investigating various anti-myeloma treatments, the 5-year survival rate for multiple myeloma (MM) patients in the US is only 55%, and almost all patients relapse. Poor patient outcomes demonstrate that myeloma cells are “born to survive” which means they can adapt and evolve following treatment. Thus, new therapeutic approaches to combat survival mechanisms and target treatment resistance are required. Importantly, Mcl-1, anti-apoptotic protein, is required for the development of MM and treatment resistance. This study looks at the possibility of KS18, a selective Mcl-1 inhibitor, to treat MM and overcome resistance. Our investigation demonstrates that KS18 effectively induces cell death in MM by dual regulatory mechanisms targeting the Mcl-1 protein at both transcriptional and post-translational levels. Specifically, KS18 suppresses Mcl-1 activation via STAT-3 pathway and promotes Mcl-1 phosphorylation/ubiquitination/proteasome-dependent protein degradation (UPS). Significantly, KS18 triggered caspase-dependent apoptosis in MM patient samples and bortezomib-resistant cells, synergizing with venetoclax to boost apoptosis. KS18 promises to overcome bortezomib and venetoclax resistance and re-sensitize myeloma cells to chemotherapy. Furthermore, the study shows the tremendous impact of KS18 in inhibiting colony formation in bortezomib-resistant cells and demonstrates significant tumor shrinkage in KS18-treated NSG mice without notable toxicity signs after 4 weeks of therapy with a single acceptable dose each week, indicating its powerful anti-neoplastic and anti-resistance characteristics. This study strongly implies that KS18 may treat MM and provide new hope to patients who are experiencing recurrence or resistance.

## Highlights

• KS18, a selective Mcl-1 inhibitor, exhibits anti-myeloma efficacy by triggering apoptosis, overcoming resistance, and enhancing sensitivity to bortezomib and venetoclax.
• KS18 shows *In vivo* significant tumor shrinkage, offering a breakthrough in multiple myeloma treatment.

## Introduction

Multiple myeloma (MM) is a bone marrow-based hematologic cancer caused by clonal plasma cell growth (Kuehl and Bergsagel, 2012; Rajkumar et al., 2014). These cells have many cytogenetic abnormalities that affect prognosis and treatment (Kumar and Rajkumar, 2018). A proteasome inhibitor, dexamethasone, and immunomodulatory or chemotherapeutic drug regimen with or without autologous stem cell transplantation is a common treatment (Moreau et al., 2011; Richardson et al., 2010; Garderet et al., 2012). These drugs are effective and often used to support other treatments, but intrinsic or acquired drug resistance makes myeloma incurable, and the disease recurs in all patients (Abdi et al., 2013).

The expression of anti-apoptotic members of the Bcl-2 family is a pivotal determinant of myeloma cell survival (Slomp and Peperzak, 2018a). The intricate interplay between anti-apoptotic (e.g., Bcl-2, Bcl-xL, Mcl-1) and pro-apoptotic (e.g., Bax, Bak, Bim, Puma, Bid, Noxa) proteins governs the apoptotic death of myeloma cells. Notably, Mcl-1 expression stands out as crucial for MM cell survival (Derenne et al., 2002; Zhang et al., 2002; Gong et al., 2016; Tiedemann et al., 2012). Indeed, anti-sense RNA suppression of Mcl-1 causes death in myeloma cells, while targeting Bcl-2 or Bcl-xL showed minimal impact or no effect (Derenne et al., 2002). In addition to the insights gleaned from *in vitro* studies highlighting the critical threshold of Mcl-1 expression required for sustaining myeloma cell viability, clinically, the overexpression of Mcl-1 is detected in 52% of MM patients at diagnosis and 81% at relapse, implying that Mcl-1 level corresponds with disease progression and correlated with a shorter lifespan (Wuilleme-Toumi et al., 2005). This highlights the potential significance of Mcl-1 as a therapeutic target in managing myeloma characterized by dysregulated intrinsic apoptosis.

Mcl-1 undergoes multifaceted regulation influencing apoptosis. Its expression is primarily controlled at the transcriptional level, with factors like STAT-3 (signal transducer and activator of transcription 3) modulating its promoter activity (Liu et al., 2003). Post-translationally, Mcl-1 stability is mediated by phosphorylation and ubiquitination, with phosphorylation at specific residues affecting protein turnover (Senichkin et al., 2020). Furthermore, protein-protein interactions with pro-apoptotic Bcl-2 family members such as Bim and Noxa also modulate Mcl-1 function, influencing apoptotic signaling (Morales et al., 2011; Gomez-Bougie et al., 2004; Dutta et al., 2010).

It has been demonstrated that anti-apoptotic proteins are attractive therapeutic targets (Punnoose et al., 2016a; Slomp and Peperzak, 2018b). Drugs that mimic the Bcl-2 homology 3 (BH3) domains of pro-apoptotic Bcl-2 family members to neutralize these proteins by attaching to their surface hydrophobic grooves are an interesting therapy option. Venetoclax (ABT-199) is the first FDA-approved Bcl-2-specific BH3 mimetic for 17p chromosomal deletion chronic lymphocytic leukemia (CLL) patients (Zhu and Almasan, 2017). Importantly, MM patients with a (11:14) translocation [t (11:14)] express more Bcl-2 than Bcl-xL or Mcl-1 and respond well to venetoclax monotherapy (Kumar et al., 2017; Cleynen et al., 2018). However, MM cells with high Mcl-1 expression are less sensitive to venetoclax (Punnoose et al., 2016a), and Mcl-1 downregulation can increase sensitivity and overcome resistance (Pandey et al., 2013; Tse et al., 2008; Cory and Adams, 2005; van Delft et al., 2006; Chen et al., 2007; Lucas et al., 2012). These findings were supported by a study on MM patients to understand their reliance on Bcl-2, Bcl-xL, and Mcl-1 using BH3 mimetics, which also showed that Bcl-2 dependency was high in a specific subgroup, while Mcl-1 dependency increased significantly from diagnosis to relapse, suggesting a shift towards Mcl-1 dependency at relapse (Gomez-Bougie et al., 2018).

Research indicates that Mcl-1 plays an essential role in both disease progression and treatment resistance. Mcl-1-targeted therapy may offer promising therapeutic avenues for MM patients, especially those experiencing relapse. Unfortunately, no FDA-approved drug specifically targets Mcl-1. To address this clinical need, our team created KS18, a pyoluteorin derivative Mcl-1 inhibitor, to test its recurrence-prevention potential (Doi et al., 2014). KS18, an effective Mcl-1 inhibitor, synergizes with existing chemotherapeutic drugs and re-sensitizes resistant MM cells to chemotherapy. This study was conducted to propose using KS18 to find novel MM therapies, especially for chemotherapy-resistant patients.

## Materials and methods

### Patient samples, cell lines, and reagents

Human MM samples were acquired from the biobank at Penn State College of Medicine, including samples from newly diagnosed patients and those who had relapsed or were refractory to treatment. Specimens were obtained after obtaining informed consent and receiving approval from the institutional review board (IRB). The malignant plasma cells were extracted from the patient samples following the established laboratory methodology (Pandey et al., 2017). MM.1S, MM.1R, U266, and RPMI 8226 human multiple myeloma cells were obtained from ATCC (Manassas, VA) and cultured in specific mediums according to our laboratory’s established technique (Elbezanti et al., 2022). MM.1S, MM.1R, and RPMI 8226 cells were grown in RPMI 1640 medium (Corning, #10-043-CV) with 10% FBS (Sigma, #F4135), and 1X of antibiotic/antimycotic solution 100X (HyClone, #SV30079.01). The U266 cells were cultivated in RPMI 1640 medium enriched with 15% fetal bovine serum (FBS) and a 1X concentration of antibiotic/antimycotic solution. The cells were cultured in a humidified incubator at a temperature of 37°C with a CO2 concentration of 5%. Dr. Nathan Dolloff from the Medical University of South Carolina in Charleston, SC kindly provided bortezomib-resistant cells (Thompson et al., 2017). Through a process of gradually increasing the dosage, we successfully created cells that are resistant to venetoclax, ABT-737, and lenalidomide. These cells are referred to as U266-VEN-R, U266-ABT-R, and U266-LEN-R. The dosage was modified at intervals of 4 weeks and/or when the cell densities doubled. The resistant cells were cultivated under identical culture conditions as their parent cells. The KS18 agent was synthesized and characterized at Penn State College of Medicine in Hershey, PA (Doi et al., 2014). All remaining agents were obtained from Selleckchem (Houston, TX).

### Cell viability assay

The cell viability was measured using the MTT assay. The MTT assay was conducted following the established protocol in our laboratory (Elbezanti et al., 2022). Overall, a total of 5,000–7,500 human MM cells were subjected to various treatments for a duration of 72 h. Twenty microliters of a recently made MTT dye solution (5 mg/mL in PBS) was added to each well, 3 h prior to the 72-h incubation. The mixture was then incubated at 37°C for 3 h. Following incubation, formazan crystals were dissolved by centrifugation of 96-well plates, removal of the supernatant, and addition of 50 µL of DMSO. The solution’s delta value (570–630 nm) was assessed using a 96-well multi-mode microplate reader from BioTek Technologies, located in Winooski, VT, United States. The cells treated with the vehicle (DMSO) were used as a control. Each treatment condition was evaluated using technical triplicates, with three wells per condition in each experiment. The average absorbance for each condition was computed, and the standard deviation (SD) was derived to evaluate the variability within the triplicate data. The results were presented as the mean value plus or minus the standard deviation. The mean, standard deviation (SD), and graph were computed and visualized using GraphPad Prism software. In order to ensure the biological reproducibility of the results, we conducted the bulk of the MTT assay in biological triplicate, which involved three separate experiments.

### Western blot

The Western blotting procedure was conducted following the way previously described (Elbezanti et al., 2022). To summarize, human MM cells were seeded at a concentration of 2 × 10^6^ cells per 1 mL of media and subjected to treatment with KS18 and/or other substances or a control solution (DMSO). The cells were then incubated at a temperature of 37°C for the prescribed period. Following incubation, whole-cell extracts were obtained by treating cells with 50 µL of lysis buffer (RIPA buffer with added protease/phosphatase inhibitor) for 1 h in an ice bucket. Following a duration of 1 h, the cells were subjected to vortexing and subsequently centrifuged at a speed of 10,000 revolutions per min for a duration of 10 min. The liquid portion was gathered, and the measurement of protein concentration was conducted using the Pierce™ BCA Protein Assay Kit (Thermo Fisher, #23225). The samples, each containing 30 μg, were subjected to separation by SDS-PAGE on NuPage 4%–12% bis-tris gradient gels (Thermo Fisher, #NP0321BOX) using either MOPS or MES solution. The proteins that had been isolated were subsequently transferred to PVDF membranes using a well-established blotting process in the laboratory. Following the transfer, the membranes were treated with 5% nonfat dry milk or BSA for 35–45 min at room temperature. This was followed by an overnight incubation at 4°C with primary antibodies. Following that, the blot was rinsed with TBST on three occasions, each lasting 5 min. Subsequently, it was subjected to horseradish peroxidase (HRP)–conjugated secondary antibodies, which were diluted at a ratio of 1:5,000, for a duration of 2 h at room temperature. Following the incubation with secondary antibodies, the membranes were rinsed with TBST solution three times for a duration of 5 min each. The membrane was treated with an enhanced chemiluminescent substrate (PierceTM ECL Western Botting, Thermo scientific, # 32106) according to the manufacturer’s instructions after the final wash. The resulting picture was captured and measured using the Bio-Rad Chemi DocTM MP imaging system. Table 1 shows that the antibodies were obtained from Cell Signaling Technology (Danvers, MA) and were used at a dilution of 1:1,000. The GAPDH antibody was employed as a loading control for all membranes. The Gentle ReView™ Stripping Buffer (VWR, #19G0856497) was employed to do several protein detections on a single membrane, thereby saving both time and samples. We performed essential blot studies with a repetition of two to three times to guarantee precision and dependability.

**TABLE 1:** List of antibodies. The following antibodies were used in this study.

Antibody	Catalog no.	Lot no.
Bad (D24A9)	9239T	5
Bak (D4E4)	12105T	4
Bax (D2E11)	5023T	2
Bcl-2 (D55G8)	4223S	6
Bcl-xL (54H6)	2764S	11
Bim (C34C5)	2933T	13
Caspase-3 (D3R6Y)	14220S	3
GAPDH (D16H11)	8884S	3
Mcl-1 (D2W9E)	94296S	5
Noxa (D8L7U)	14766T	4
PARP (46D11)	9532S	10
P-Mcl-1 (S159/T163)	4579S	5
Puma (D30C10)	12450T	5
Ubiquitin (E412J)	43124S	3
STAT3 (124H6)	9139S	16
P-STAT3 (Y705) (D3A7)	9145S	43

### Immunoprecipitation (IP)

We performed immunoprecipitation following our laboratory protocol as previously described (Pandey et al., 2013). In this experiment, human MM cells were plated at a density of 5 × 10^6^ cells per 1 mL of medium and treated with KS18. The cells were then incubated at 37°C for 24 h. The whole cell extract was incubated with either Mcl-1 or Bim antibodies overnight at 4°C. Following incubation, 20 µL of agarose A/G (Santa Cruz Biotechnology #sc-2003) was added and left to incubate at 4°C for an additional 3 h. The pellet underwent three rinses with lysis solution before being resuspended in 20 µL SDS dye for further western blotting as described above. The main immunoprecipitation experiments were repeated several times to ensure repeatability.

### Chromatin immunoprecipitation (ChIP)

The ChIP analysis process was conducted using the previously reported approach (Pandey et al., 2014; Elbezanti et al., 2020). In summary, U266 cells were placed in flasks at a density of approximately 30 × 106 cells per flask and exposed to KS18 or left untreated for 24 h at a temperature of 37°C. The cells were treated with 4% paraformaldehyde and collected using the Pierce ™ Magnetic ChIP kit (Thermo Fisher Scientific, #26157) according to the instructions provided by the manufacturer. The ChIP assays were conducted by incubating the chromatin in a buffer containing 1% triton X-100, 0.1% deoxycholate, 1x TE, and a protease inhibitor. This mixture was then combined with 20 mg of affinity-purified rabbit polyclonal anti-STAT3 antibody (Cell signaling, #9139) or normal rabbit IgG (Abcam, ab46540) as a control. The antibodies were precoated onto Goat-anti-rabbit IgG Dynabeads (Invitrogen). After being incubated overnight at a temperature of 4°C, the protein/DNA complexes were collected using a Magnetic Particle Concentrator (Invitrogen). The beads were rinsed eight times with 1 mL of RIPA buffer (composed of 50 mM Hepes at pH 8.0, 1 mM EDTA at pH 8.0, 1% NP-40, 0.7% deoxycholate, and 0.5 M LiCl) and then finally with 1 mL of TE. Following the extraction of TE, the DNA was separated using 50 mL of elution solution containing 10 mM Tris pH 8.0, 1 mM EDTA, and 1% SDS. The crosslinks were disrupted in the presence of a 0.6 M NaCl solution at a temperature of 65°C for the duration of one night. The samples were treated with a proteinase K Mix (consisting of 14 mg protease K and 3 mg glycogen in 1x TE) for a duration of 2 h at a temperature of 37°C. Following this, the samples were extracted using a phenol/chloroform solution. Subsequently, the samples were treated with 10 mg of RNase A for a duration of 2 h at a temperature of 37°C. Finally, the DNA was obtained using the QIA quick PCR Purification kit (manufactured by QIAGEN). The ChIP sample was assessed for enrichment over the input by doing qPCR with particular primers in the promoter region of target genes. This was done using an AB Applied Biosystems Step One Pulse Real-Time PCR System and TaqMan universal PCR Master Mix (Applied Biosystems, Foster City, CA). The housekeeping gene was utilized for delta-delta Ct (ΔΔCt) computations. The data were examined utilizing the specified method and then standardized in relation to the input for each condition. The cells treated with the vehicle served as the control group for comparison. The data were shown as the mean value plus or minus the standard error of measurement.

### Cytochrome C release test

The human MM cells were treated with or without KS18 and incubated for 24 h at 37°C. Following incubation, the cytosolic fraction was extracted using the cytochrome C release assay kit (Abcam, #ab65311) according to the manufacturer’s instructions. Protein quantitation and western blotting were performed using the previously published method (Elbezanti et al., 2022).

### Annexin V live dead assay

Human MM cells were plated, treated, and incubated for 24 h, and then tested for Annexin V Live Dead assay (Luminex Corporation, #MCH100105). Cells were stained with 1:1 Annexin V at room temperature in the dark for 20 min following manufacturer’s protocol. Data was analyzed using Muse^®^ Cell Analyzer (Elbezanti et al., 2022). Vehicle treated cells served as controls. Each treatment condition was assessed using three technical triplicates, with three wells per condition in each experiment. The early and late apoptosis were combined to get the overall apoptosis. The data were reported as the mean value ± standard deviation. The mean and standard deviation (SD) were calculated, and a graph was created using GraphPad Prism software. To guarantee the biological repeatability of the data, we conducted the apoptotic experiment multiple times.

### Caspase-3/7 assay

Human MM cells were subjected to treatment with or without experimental substances and then incubated for a duration of 24 h. Afterward, the cells were examined for the presence of caspase 3/7 using a kit provided by Luminex Corporation (# MCH100108). The cells were subjected to staining with caspase 3/7 working solution for a duration of 30 min at a temperature of 37°C, in accordance with the instructions provided by the manufacturer. The 7-AAD solution was added subsequent to incubation, and the caspase assay was completed. The data was evaluated using the Muse^®^ Cell Analyzer (Elbezanti et al., 2022). Vehicle treated cells were used as controls. The statistics were given as the average value plus or minus the standard deviation. The mean and standard deviation (SD) were computed, and a graph was generated using GraphPad Prism software. Every experiment was replicated three times to determine the standard deviation and verify the correctness and dependability of the findings.

### Flow cytometry

MM patient samples or human MM cells were plated, treated, and incubated for 24 h before being tested using Annexin V Apoptosis Detection Kit (BD Biosciences, #556547). Annexin V/PI stain solution was combined with cells and incubated at room temperature in the dark for 20 min before detection of dead cells using flow cytometer (BIO-RAD, Se™ Cell Sorer) (Elbezanti et al., 2022). Vehicle treated cells served as control. Percentage of total apoptosis was estimated as [(% cell death in treated cells − % cell death in control)/(% viable cells control) ×100].

### Immunostaining and confocal microscopy

The immunostaining procedure was carried out following the previously described method (Elbezanti et al., 2022). MM cells were plated and treated with KS18 for 24 h. The cell suspension was carefully loaded into the funnel designed for the Cytospin. The cells underwent centrifugation for 3 min at 800 rpm and were then fixed with 4% formaldehyde for a duration of 10 min. Following three rounds of washing with ice-cold PBS, the cells were then incubated with methanol for a duration of 5 min. The sample was blocked with 1% BSA for 1 h, followed by the addition of a 1:200 dilution of the primary antibody in 1% BSA. The mixture was then incubated overnight at 4°C. The sample was washed three times with PBS, followed by the addition of the secondary antibody goat anti-rabbit IgG (H + L) Alexa FluorTM Plus 488 (Invitrogen, #A32731) at a dilution of 1:400. The mixture was gently rocked for 1 h at room temperature. The slides were mounted using ProLongTM Diamond Antifade Mount with DAPI (Invitrogen, #P36971) and a coverslip was placed on top. The photograph was captured using a Nikon A1R GaAsP Laser Scanning Confocal Microscope (Nikon Eclipse Ti inverted with a 60x objective). Vehicle treated cells served as control. NIH ImageJ software was used to analyze the samples.

### Colony forming assay

The colony forming assay was performed essentially following the manufacturer’s protocol. Briefly, human MM cells were cultured at a density of 1 × 10^4^ cells in MethoCult™ H4230 media (Stem Cell Technologies, #04230) supplemented with 15% FBS and 10% Gibco Phytohemagglutinin M form (PHA-M) (Thermo Fisher, #10576015) stimulated leukocyte conditioned medium. Human MM cells were incubated at 37°C in 5% CO2 for 14 days before colonies were visually counted using a microscope. Vehicle treated cells served as control. The experiments were performed in triplicates. The survival fraction (SF) was determined by dividing the number of colonies in the treated group by the number of colonies in the control group. The statistical analysis (e.g., t-test or ANOVA) was performed to assess the significance of KS18’s effects on colony formation.

### Virus titration and Mcl-1 human shRNA lentiviral transduction

A total of 100 µL of human MM cells was distributed into each well of a 96-well flat bottom plate at a density of 1 × 105 cells per well using a multi-pipette channel. Subsequently, 100 μL of heated medium was introduced into every well of a 96-well round bottom plate, and then 50 μL of the OriGene virus (MCL1 Human shRNA Lentiviral Particle #Locus ID 4170) was added to well A of the same plate. The materials were thoroughly mixed prior to performing serial dilution, beginning with the transfer of a 50 μL solution from well A to well B, continuing through well G, and leaving well H devoid of any virus. The multichannel pipette was used to transfer 50 μL of each dilution of the viral suspension from the titer plate to the flat bottom plate that was prepared on Day 0. Human MM cells were cultured at 37°C in a 5% CO2 environment for 72 h. After incubation, the cells were examined under a microscope to assess the intensity of GFP fluorescence. The most suitable dilution was selected, and the cells were treated with puromycin (2 μg/mL) for 7 days. The concentration of puromycin was determined by conducting a toxicity curve to identify the most effective concentration for our experiment. The cells that were chosen with puromycin were employed immediately for the studies. The silencing impact of the shRNA constructs after transfection was assessed using western blot. To accurately evaluate knockdown, the gene expression level of Mcl-1 was measured and compared to the control vector with scrambled sequence.

### Toxicity and efficacy studies in mice xenograft model

The five-week-old female NOD-SCID-IL2R gamma null (NSG) mice were obtained from Jackson Laboratories and carefully maintained and monitored at the animal research facility located at Cooper Medical School of Rowan University (CMSRU) in Camden, NJ. The xenograft investigations were carried out at CMSRU in compliance with the ethical guidelines set by Rowan University’s Institutional Animal Care and Use Committee (IACUC). For the experiment, 5 × 10^6^ U266 MM cells in a 1:1 ratio of U266 and Matrigel basement membrane matrix were injected into the right flank of the mice to establish human MM xenografts. After palpable tumors (volume ∼100 mm^3^) appeared approximately 10 days post-injection, the animals were randomly divided into three groups of five mice each, including a vehicle control group. The mice were administered weekly treatments of either DMSO or KS18 (at doses of 5 and 10 mg/kg) for a duration of 4 weeks by intraperitoneal injections. For quantifying tumor volume, we took digital caliper measurements of the longest perpendicular tumor diameter in the following manner: Tumor volume = 4/3 π × (width/2)^2^ × (length/2). The tumor volume and body weight were measured once a week. At the conclusion of the study, the animals were compassionately euthanized, and tumors were then carefully extracted, weighed, and promptly frozen to facilitate future research.

The sample size for this investigation was calculated using power calculations to ensure the ability to detect statistically significant changes between experimental groups. The study selected a sample size of 5 mice per group, which ensures that it has enough statistical power to detect significant effects in tumor growth that are biologically important. The study has a power of 80% (β = 0.2) and a significance level (α) of 0.05. Given an anticipated disparity of at least 30% in tumor size between groups, with a standard deviation of 20%, a power analysis utilizing a two-tailed t-test has determined that a sample size of 5 mice per group would be adequate to detect this magnitude of effect with the specified power and significance level. This sample size also addresses ethical concerns by minimizing the number of animals necessary to obtain reliable results, while still preserving sufficient statistical validity.

### Statistical analysis

Statistical significance of the results was analyzed by unpaired two-tailed Student’s t-test, one-way analysis of variance (ANOVA), or two-way ANOVA using GraphPad prism software. Each graph represents the average of at least three replicates with error bars on the graph representing standard deviation *P ≤ 0.05, **P ≤ 0.01, ***P ≤ 0.001, ****P ≤ 0.0001. ns: non-significant.

## Results

### KS18 inhibits Mcl-1 selectively in MM cells

**FIGURE 1:**
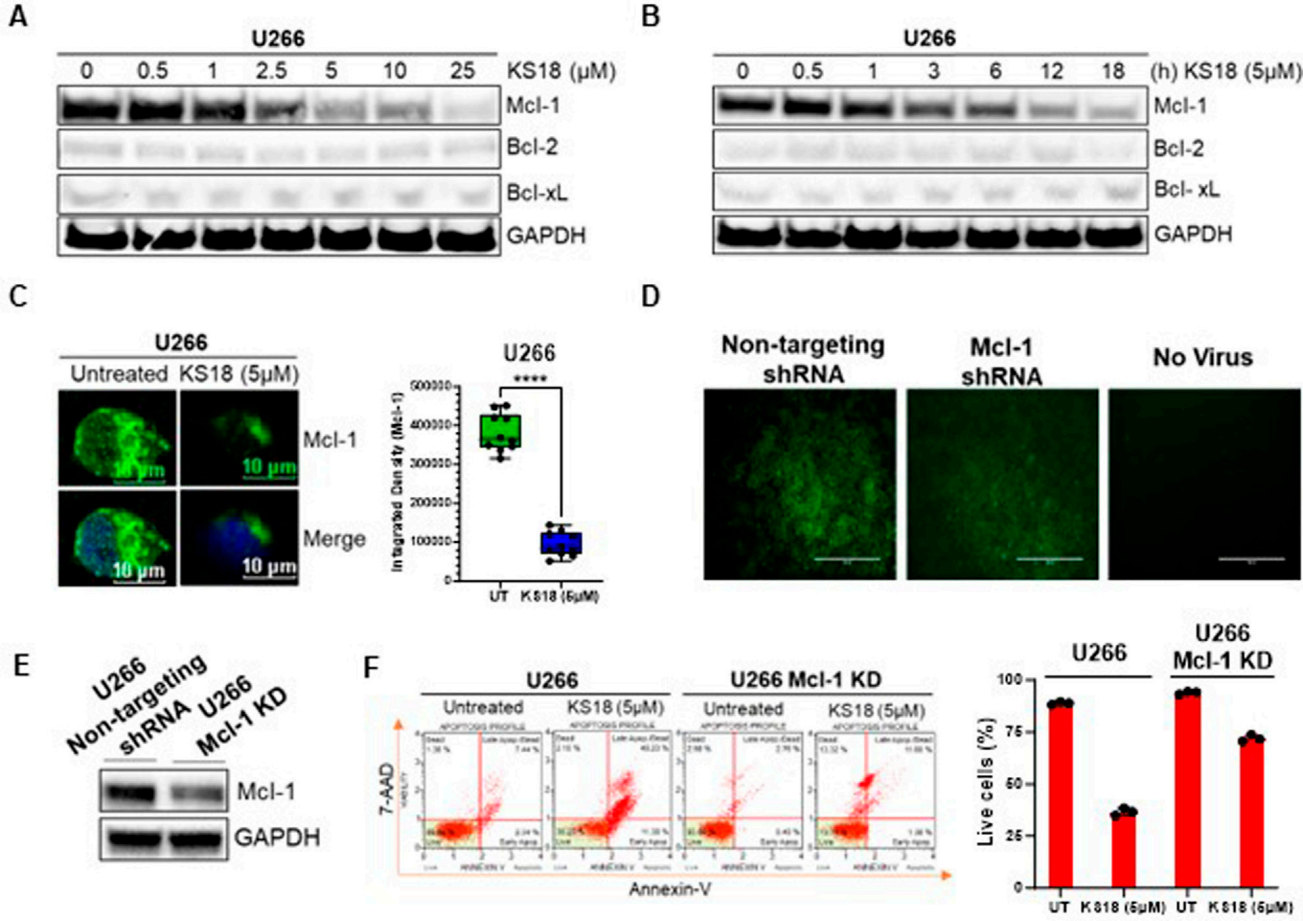
Effect of KS18 on Mcl-1 in MM cells. **(A, B)**, U266 MM cells were treated with or without specified doses of KS18 for 24 h, or with 5 µM of KS18 at the designated time intervals **(B)**. **(C)**, U266 MM cells were subjected to treatment with or without 5 μM of KS18 for a duration of 24 h. Subsequent to incubation, immunocytochemical analysis was conducted as outlined in the Materials and Methods section. ImageJ software was utilized to compute the integrated density of individual samples (n = 10), and an unpaired t-test was conducted using GraphPad Prism. **(D)**, The selection of Mcl-1 shRNA or non-targeting shRNA transfected cells. **(E)**, Western Blot was used to evaluate the silencing effect of the shRNA constructs. The non-targeting shRNA transfected cells served as control. **(F)**, U266 and U266 Mcl-1 knockdown cells were administered KS18 (5 µM) for 24 h, after which an apoptotic test was conducted as outlined in the Materials and Methods section utilizing the Muse^®^ Cell Analyzer. For section **(A, B, E)**, subsequent to incubation, the cells were lysed and analyzed via immunoblotting as outlined in the Materials and Methods section, utilizing the specified antibodies. The GAPDH antibody functioned as a loading control. Cells treated with vehicles served as the control. For section **(F)**, GraphPad Prism was utilized to generate graphs and statistical calculations. ****P ≤ 0.0001.

KS18 agent was synthesized and characterized at Penn State College of Medicine, Hershey, PA (Doi et al., 2014). We used X-ray crystal structure of Mcl-1 from the Protein Data Bank (PDB ID: 2JM6, 2.5 Å) to perform docking studies using Autodock Vina program. Docking results suggest that KS18 interacts with Mcl-1 (Dominguez et al., 2003). The protein-protein restraint docking of NOXA with Mcl-1 using the HADDOCK (high ambiguity-driven protein-protein docking) algorithm confirmed that KS18 binds Mcl-1 at its NOXA binding sites (Supplementary Figure S1A). We next determined the expression of anti-apoptotic family members in MM cell lines. Most myeloma cells, except RPMI 8226, overexpress Mcl-1, suggesting these cells are dependent on Mcl-1 for survival. U266 cells showed maximum expression of Mcl-1, followed by MM.1S and MM.1R. RPMI 8226 cells, on the other hand, are likely dependent on Bcl-2 and Bcl-xL (Supplementary Figure S1B). Due to the fact that U266 cells exhibited a reliance on Mcl-1, we opted to conduct further investigations using U266 cells. Supplementary Material display the results of all experiments conducted on additional MM cell lines, namely MM.1S and MM.1R. We further investigated the effect of KS18 on Mcl-1. As shown in Figures 1A, B, KS18 inhibits Mcl-1 expression in a dose- and time-dependent manner, but importantly, did not demonstrate any effect on Bcl-2/Bcl-xL expression. Similarly, KS18 was effective at lowering Mcl-1 expression in additional MM cell lines (MM.1S, and MM.1R; Supplementary Figure S1C). Immunocytochemical analysis using confocal microscopy also suggested that KS18 inhibits Mcl-1 expression in U266 MM cells (Figure 1C). Because KS18 off-targets can lead to unexpected side effects and to ensure the selectivity of KS18, the study utilized Mcl-1 human shRNA lentiviral particle to knock down Mcl-1 expression. The knockdown efficiency was evaluated using GFP tagging and western blot analysis. The infected cells were chosen based on their expression of GFP, and then subjected to puromycin selection at a concentration of 2 μg/mL (Figure 1D). The silencing impact of the shRNA constructs post-infection was assessed using Western blot. In order to accurately evaluate knockdown, the level of Mcl-1 was examined in Mcl-1 KD cells and compared to the cells infected with a scramble vector. Figure 1E illustrates the reduction of Mcl-1 protein levels in U266 Mcl-1KD cells following infection with Mcl-1 human shRNA lentivirus. After verifying the efficacy of Mcl-1 human shRNA lentiviral in suppressing Mcl-1, we examined the impact of KS18 on apoptosis in U266 and U266 Mcl-1KD cells. Figure 1F demonstrates that KS18 is very efficient in cells that express Mcl-1, but it becomes less effective in cells with reduced Mcl-1 levels (Mcl-1KD). This suggests that the ability of KS18 to kill cells is dependent on the presence of Mcl-1.

### KS18 targets the STAT-3/Mcl-1 axis and facilitates Mcl-1 degradation

**FIGURE 2:**
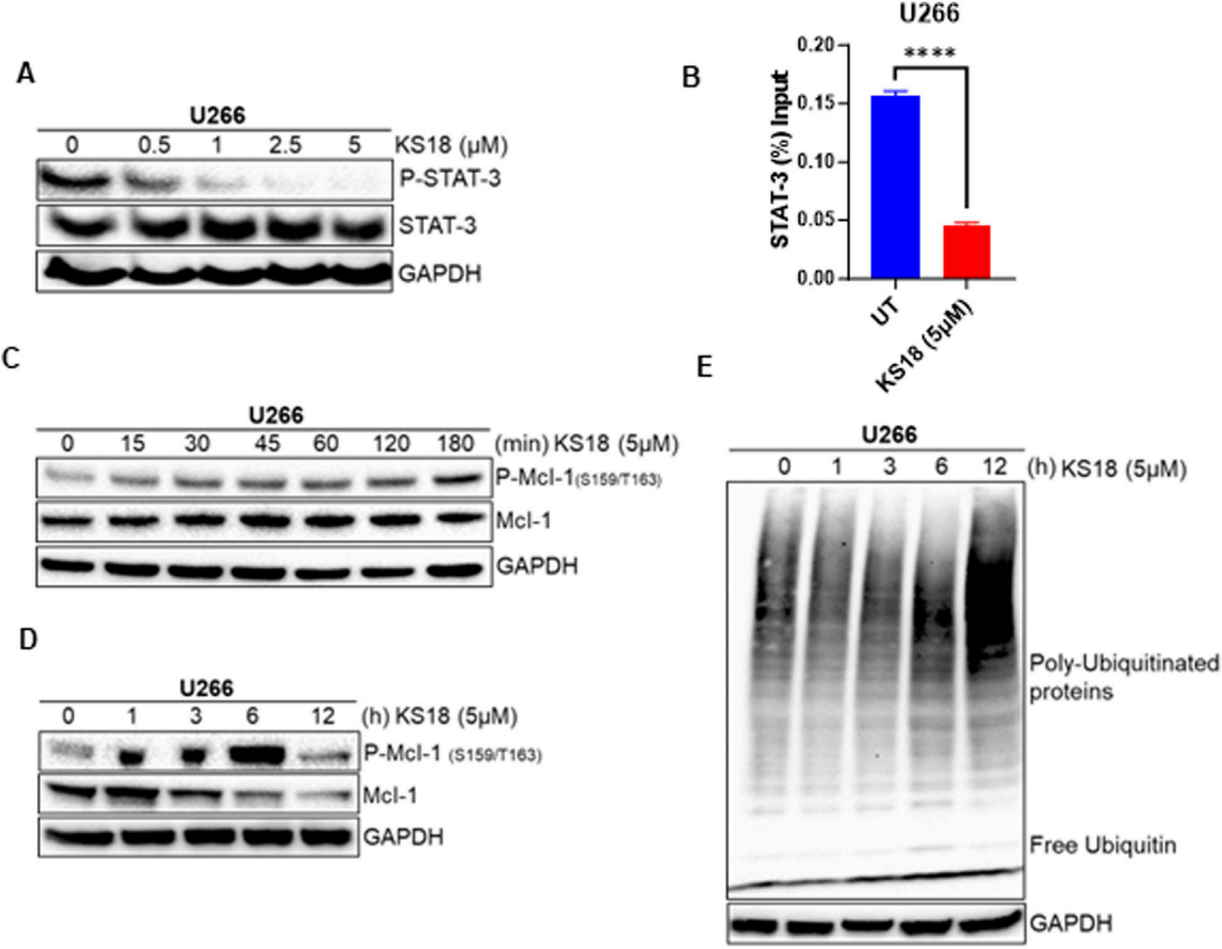
KS18 inhibits Mcl-1 at both transcriptional and post-translational levels. **(A)**, U266 MM cells were treated with or without the indicated doses of KS18 for 24 h **(B)**, KS18 therapy suppresses the activation of the STAT3-Mcl-1 promoter. The ChIP experiment was conducted as outlined in the Materials and Methods section. GraphPad Prism was utilized for graphical representations and statistical analysis. All data were shown as mean ± standard error of measurement. **(C–E)**, 5 μM of KS18 was applied to U266 MM cells at various time points. For section **(A, C, D, E)**, subsequent to incubation, the cells were lysed and analyzed via immunoblotting as outlined in the Materials and Methods section, utilizing the specified antibodies. The GAPDH antibody functioned as a loading control. Cells treated with vehicles served as the control. ****P ≤ 0.0001.

We investigated the effects of KS18 on Mcl-1 regulation, focusing on transcriptional and post-translational modifications and their impact on protein stability. The transcription factor STAT-3 plays a crucial role in MM by being constantly activated and promoting the survival of cancerous cells through the regulation of the anti-apoptotic protein Mcl-1 (Liu et al., 2003; Chong et al., 2019). Therefore, blocking the STAT3-Mcl-1 pathway in cancer has been demonstrated to reduce Mcl-1 levels, which in turn promotes programmed cell death in cancer cells. This presents a promising therapeutic approach for specifically targeting malignancies (Kiprianova et al., 2015). We examined the effect of KS18 on phosphorylation of STAT3 in U266 cells. KS18 demonstrated dose-dependent inhibition of phosphorylated STAT-3 (P-STAT-3) protein, without effecting the expression of total STAT3, indicating its ability to modulate STAT-3 activation (Figure 2A). Remarkably, our chromatin immunoprecipitation (ChIP) analysis revealed that KS18 inhibits STAT-3 binding to the Mcl-1 promoter, resulting in diminished Mcl-1 transcriptional activation (Figure 2B). This observation underscores the importance of STAT-3 as a key transcriptional regulator of Mcl-1 expression and highlights the specificity of KS18 in targeting this pathway. At the post-translational level, Mcl-1 degradation is controlled by phosphorylation at the Ser159/Thr163 sites, which leads to ubiquitination by E3 ligases like F-box and WD repeat domain-containing 7 (FBW7), Mule, and b-TrCP (Senichkin et al., 2020; Zhong et al., 2005). In order to investigate the mechanism by which KS18 degrades Mcl-1, we conducted experiments to examine the impact of KS18 on the phosphorylation and ubiquitination of Mcl-1. As depicted in Figure 2C, KS18 triggers the phosphorylation of Mcl-1 at the Ser159/Thr163 sites, with this process commencing as early as 15 min after treatment. The maximum level of phosphorylation occurs within 6 h of treatment, as shown in Figure 2D. Remarkably, the decline of Mcl-1 was found to be associated with the phosphorylation of Mcl-1. According to Figure 2D, the breakdown of Mcl-1 begins 3 h after exposure to KS18. The degradation of Mcl-1 was associated with the induced ubiquitination process. Figure 2E demonstrates the degradation of Mcl-1 through ubiquitination throughout time. The highest level of ubiquitination was seen at 12 h, which corresponds to the destruction of Mcl-1 (Figures 2D, E). Our results demonstrate that treatment of KS18 inhibiting Mcl-1 by both transcriptionally, via disrupting STAT3-Mcl-1 promoter activation (Figure 2B), and post-translationally, via inducing phosphorylation of Mcl-1 as early as 15 min followed by its ubiquitination (Figures 2C–E). Taken together, this finding indicates that KS18 plays a dual role at both transcriptional and post-translational levels of Mcl-1 inhibition and this dual mechanism of action contributes to its efficacy in inducing cell death in MM. However, it is unclear to us which mechanism is more dominant. It is worthwhile to investigate how KS18 regulates two distinct processes.

### KS18 induces apoptosis in a caspase-dependent manner

**FIGURE 3:**
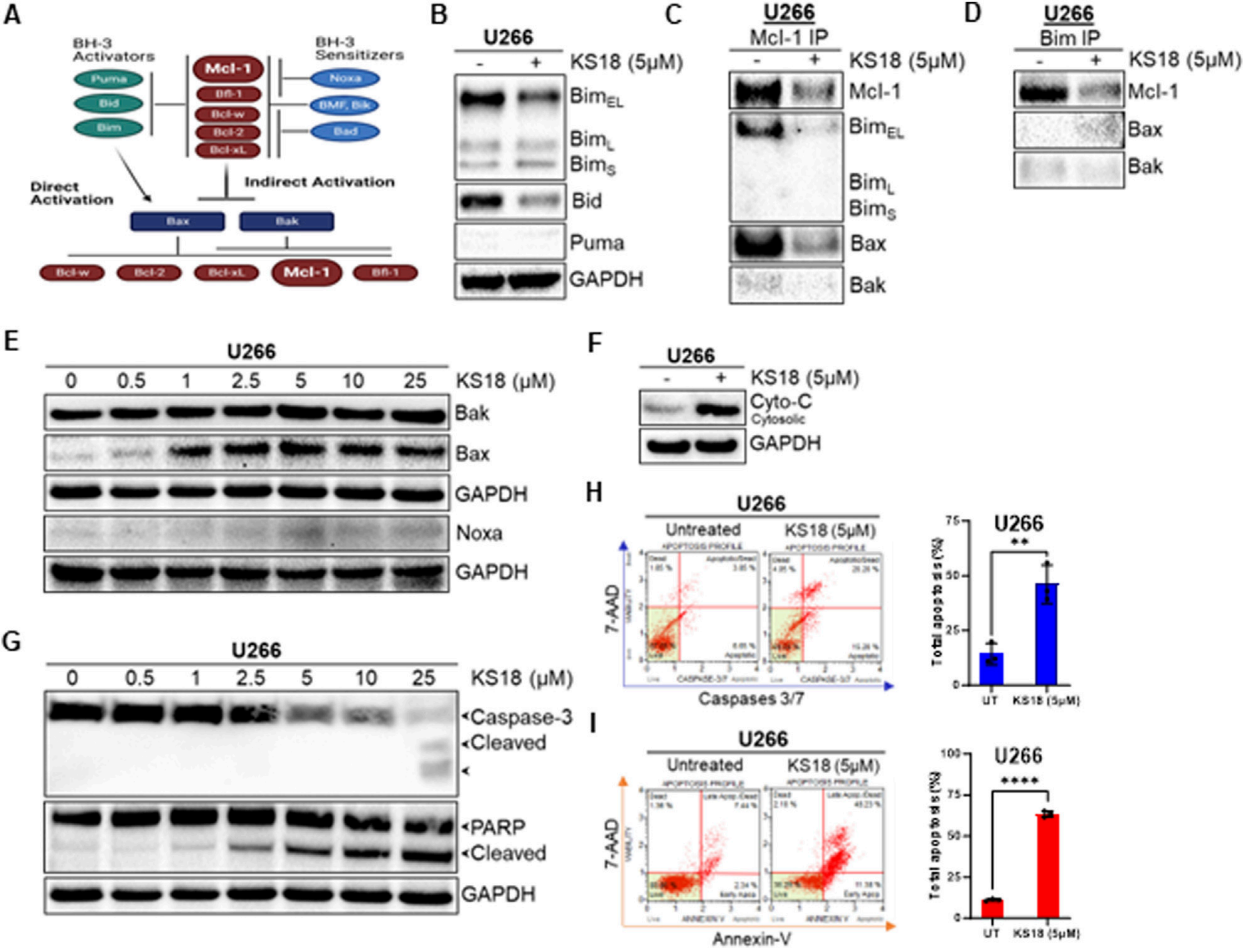
Effect of KS18 on apoptosis regulators. **(A)**, The schematic presentation of apoptotic regulators. In the indirect activation, upregulation of BH3-only proteins will act as inhibitors of anti-apoptotic proteins by competing for their binding with Bax and Bak proteins, leading Bax and Bak to oligomerize. In the direct activation, upregulation of BH3 activators proteins directly activates Bax and Bak. **(B)**, U266 MM cells were treated with or without KS18 (5 µM) for 24 h. **(C, D)**, an immunoprecipitation test was conducted to evaluate the interaction between Mcl-1 and Bim, Bak, and Bax, utilizing either anti-Mcl-1 or anti-Bim for the pull-down. Immunoprecipitation followed by western blotting was conducted as outlined in the Materials and Methods section. **(E–G)**, U266 MM cells were treated with the indicated doses of KS18 for 24 h. Specific to section **(F)**, after incubation the cytosolic fractions were isolated using a cytochrome C kit. **(H, I)**, Caspases 3/7 and Annexin V staining were used to identify apoptotic cells. U266 cells were treated with KS18 (5 µM) for 24 h before being stained with caspases 3/7 dye or Annexin V dye as described in Materials and Methods section and evaluated using the Muse^®^ Cell Analyzer. The total number of apoptotic cells was enumerated for samples (n = 3) and unpaired t test was performed using GraphPad prism software. In all experiments, vehicle treated cells served as control. For sections **(B–G)**, after incubation, the cells were lyzed and immunoblotting was performed using the mentioned antibodies. **P ≤ 0.01, ****P ≤ 0.0001.

A network of pro-and anti-apoptotic proteins controls cell destiny (Figure 3A). BH3 domains of pro-apoptotic proteins bind to anti-apoptotic proteins’ hydrophobic groove. In Mcl-1-expressing myeloma cells, Bim distribution dictates Mcl-1 dependency or codependence with Bcl-2/Bcl-xL (Morales et al., 2011; Gomez-Bougie et al., 2004). The binding of Mcl-1 to pro-apoptotic protein Bim was determined. Figure 3B shows that after 24 h KS18 lowered Bim_EL_ but not Bim_L_ or Bim_S_. Bid expression was likewise decreased in KS18-treated cells. Using immunoprecipitation, we pulled down Mcl-1 and Bim to explore their relationship. In KS18-treated cells, after 24 h, reduced Mcl-1 expression was related to poor binding of Bim_EL_ followed by Bax (Figure 3C). We also found similar results when we pulled down Bim followed by Mcl-1 immunoblotting (Figure 3D), demonstrating that Bim dissociates from Mcl-1 and activates Bax. Therefore, Bax and Bim are the major mediators of KS18-induced apoptosis via Mcl-1 inhibition. Similarly, the administration of KS18 leads to an increase in Bax expression and does not have any impact on Bak (Figure 3E). This indicates that Bax acts as the mediator of apoptosis produced by KS18 through the degradation of Mcl-1. Furthermore, we examined the impact of KS18 on Noxa protein. KS18 had no affect on Noxa expression (Figure 3E). Bax and Bak oligomerize and produce pores in the outer mitochondrial membrane, releasing cytochrome C and other apoptogenic factors into cytosol (Dewson and Kluck, 2009). Since KS18 therapy activates Bax (Figure 3E), we explored if it releases cytochrome C. After 24 h, KS18 treatment released cytochrome C into the cytosol (Figure 3F). Subsequently, in U266 cells, KS18 stimulates caspase-3 cleavage via procaspase-3 processing and PARP cleavage (Figure 3G). We verified this in additional MM cell lines, MM.1S and MM.1R (Supplementary Figure S1D). KS18’s apoptosis-inducing capacity in MM cells was examined by caspase-3/7 and Annexin V staining. KS18 substantially causes caspase-dependent MM cell death (Figures 3H, I; Supplementary Figures S1E, F). These data suggest that KS18 may induce intrinsic apoptosis in MM cells via promoting Bax-induced mitochondrial permeabilization, cytochrome C release, and caspase activation followed by PARP cleavage.

### KS18 demonstrates significant effectiveness as a standalone treatment in MM cell lines and patient samples, while also boosting the cytotoxic impact of bortezomib and venetoclax in MM cells

**FIGURE 4:**
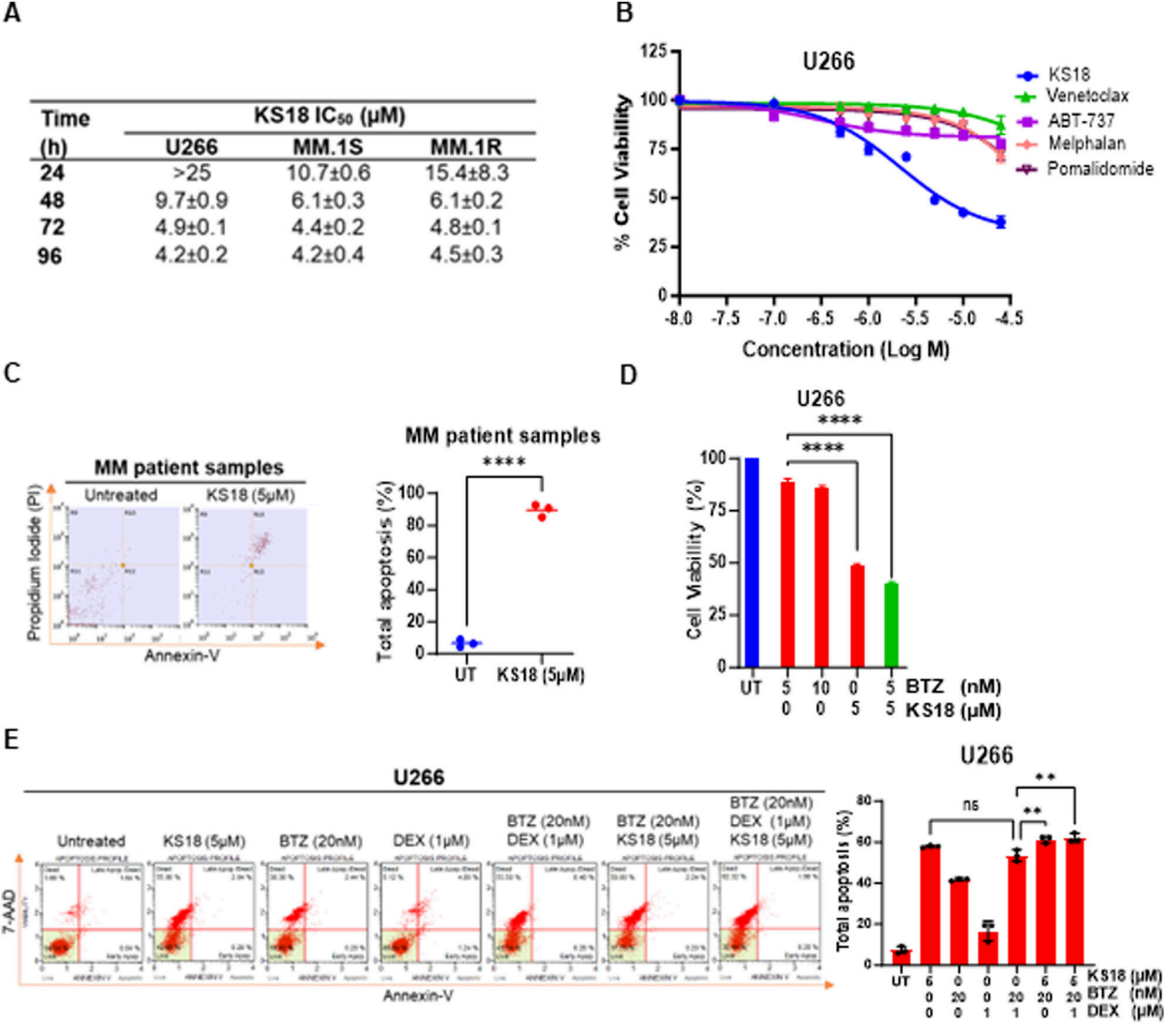
KS18 is effective against MM cells, synergize with bortezomib and causes apoptosis in MM patients. **(A)**, A panel of human MM cell lines (U266, MM.1S, and MM.1R) were treated with increasing doses of KS18 (0–25 μM) for 24, 48, 72, and 96 h, and the cytotoxicity of KS18 was determined using the MTT assay. The IC_50_ values were calculated using GraphPad prism. **(B)**, U266 cells were subjected to escalating concentrations (0–25 μM) of KS18 and several chemotherapeutic agents (venetoclax, ABT-737, melphalan, and pomalidomide) for 72 h, after which cell viability was evaluated using the MTT assay. **(C)**, MM patient samples (n = 3) were subjected to treatment with or without KS18 (5 µM) for a duration of 24 h. The Annexin V Apoptosis Detection Assay was conducted via FACS. The percentage of total apoptosis was calculated as [(% cell death in treated cells − % cell death in control)/(% viable cells in control) × 100]. **(D)**, U266 cells were subjected to a 72-h treatment with bortezomib (BTZ) at concentrations of 5 and 10 nM, either alone or in conjunction with KS18 at 5 μM, and cell viability was assessed using the MTT test. **(E)**, U266 cells were subjected to a 24-h treatment with KS18 (5 µM) either alone or in conjunction with BTZ (20 nM) and dexamethasone (DEX) (1 µM), thereafter stained with Annexin V dye and evaluated by the Muse^®^ Cell Analyzer. The total count of apoptotic cells was quantified for samples (n = 3), and one-way ANOVA was conducted utilizing GraphPad Prism software. In all experiments cells treated with vehicle served as the control group. GraphPad Prism was utilized to generate graphs and statistical analysis. ****P ≤ 0.0001, **P < 0.01, ns, not significant.

An MTT assay was conducted to assess the cytotoxic impact of KS18 on the viability of cancer cells. This evaluation was performed on three MM cell lines (U266, MM.1S, and MM.1R) at 24, 48, 72, and 96 h (Figure 4A). The IC_50_ values in MM.1R fell from over 15.4 ± 8.3 μM at 24 h to 4.5 ± 0.3 μM at 96 h. In the MM.1S cell line, the IC_50_ values decreased from 10.7 ± 0.6 µM to 4.2 ± 0.4 µM. Similarly, the U266 cells exhibited the highest sensitivity, with IC_50_ values dropping from more than 25 µM to 4.2 ± 0.2 µM throughout the same time frame (Figure 4A). The results demonstrate that the effectiveness of KS18 increases with longer exposure times and is reliant on the expression of Mcl-1. KS18’s cytotoxic capability was compared to other chemotherapy drugs. A 72-h MTT experiment demonstrated that the KS18 exhibited superior performance compared to all tested chemotherapeutic drugs in lowering cell viability in U266 cells (Figure 4B). The IC_50_ of the KS18 was markedly lower compared to the chemotherapeutic drugs, suggesting a higher level of cytotoxic effectiveness (Figure 4B). The data indicate that KS18 is superior to traditional chemotherapeutic treatments in suppressing cell proliferation in U266 cell line and other Mcl-1 expressing cell lines (Figure 4B; Supplementary Figures S2A–C). In addition, we conducted tests to evaluate KS18’s effectiveness in inducing apoptosis in MM samples. The flow cytometry analysis using Annexin V/PI showed that the KS18 therapy (5 µM for 24 h) had a significant impact on MM patient samples, leading to a high rate of apoptosis 88.8%, as shown in Figure 4C. We explored the potential of combining KS18 with bortezomib, a commonly used first-line therapy. Figure 4D and Supplementary Figure S2D suggest that KS18 enhances the therapeutic efficacy of bortezomib. The combination of KS18 (5 µM) and bortezomib (5 nM) significantly reduces MM cell viability by 56%–70%.

The clinic recommends the use of dexamethasone and either bortezomib or lenalidomide for individuals with standard-risk myeloma (Durie et al., 2017). If rescue regimens fail and the malignancy grows resistant, cyclophosphamide, melphalan, or doxorubicin can be included as additional treatment options (San Miguel et al., 2008; Kumar et al., 2012; Harousseau et al., 2010). We evaluated KS18 with dexamethasone and bortezomib for clinical simulation. KS18 in combination with bortezomib and low-dose dexamethasone improves therapeutic response, suggesting that KS18 may improve therapeutic outcomes (Figure 4E). A similar impact was seen in MM.1S cells (Supplementary Figure S2E).

Although venetoclax is a selective small-molecule Bcl-2 inhibitor, it is ineffective against cancer cells with high Mcl-1 levels, including MM (Phillips et al., 2015; Bodo et al., 2016; Konopleva et al., 2016; Punnoose et al., 2016b). We tested whether KS18 sensitizes Mcl-1 overexpressing cells to venetoclax by treating MM cells with either KS18 or venetoclax alone or in combination. The treatment of venetoclax alone induces the expression of Mcl-1, but it was suppressed when treated with both KS18 and venetoclax (Supplementary Figure S3A). Similarly, this was observed in other MM cells (Supplementary Figure S3B). Furthermore, the apoptosis-inducing potential of KS18 in combination with venetoclax was investigated. In U266 cells, the addition of KS18 (5 µM) to venetoclax (0.5 µM) exhibited a strong additive impact, with significant stimulation of caspase-mediated apoptosis (Supplementary Figures S3C, D). The same response was observed in MM.1S cells (Supplementary Figures S3E, F). Furthermore, the addition of KS18 (5 µM) to venetoclax resulted in a considerable reduction in cell viability in MM cells (Supplementary Figure S3G).

We investigated whether ABT-737 (small-molecule Bcl-2/Bcl-xL inhibitor) had similar effect as was observed with venetoclax. As expected, ABT-737 alone increased Mcl-1 expression, while KS18 and ABT-737 suppressed Mcl-1 (Supplementary Figures S4A, B). The apoptosis-inducing ability of KS18 in conjunction with ABT-737 was confirmed (Supplementary Figures S4C–F). As observed with venetoclax, adding KS18 (5 µM) to ABT-737 significantly reduced cell viability in MM cells (Supplementary Figure S4G). Our research shows that inhibiting Mcl-1 is necessary to enhance the effectiveness of venetoclax and ABT-737 in treating diseases. Therefore, the effectiveness of Bcl-2/Bcl-xL inhibitors in MM cells can be enhanced by including the Mcl-1 inhibitor KS18.

### KS18 re-sensitizes bortezomib-resistant MM cells and kills resistant MM cells

**FIGURE 5:**
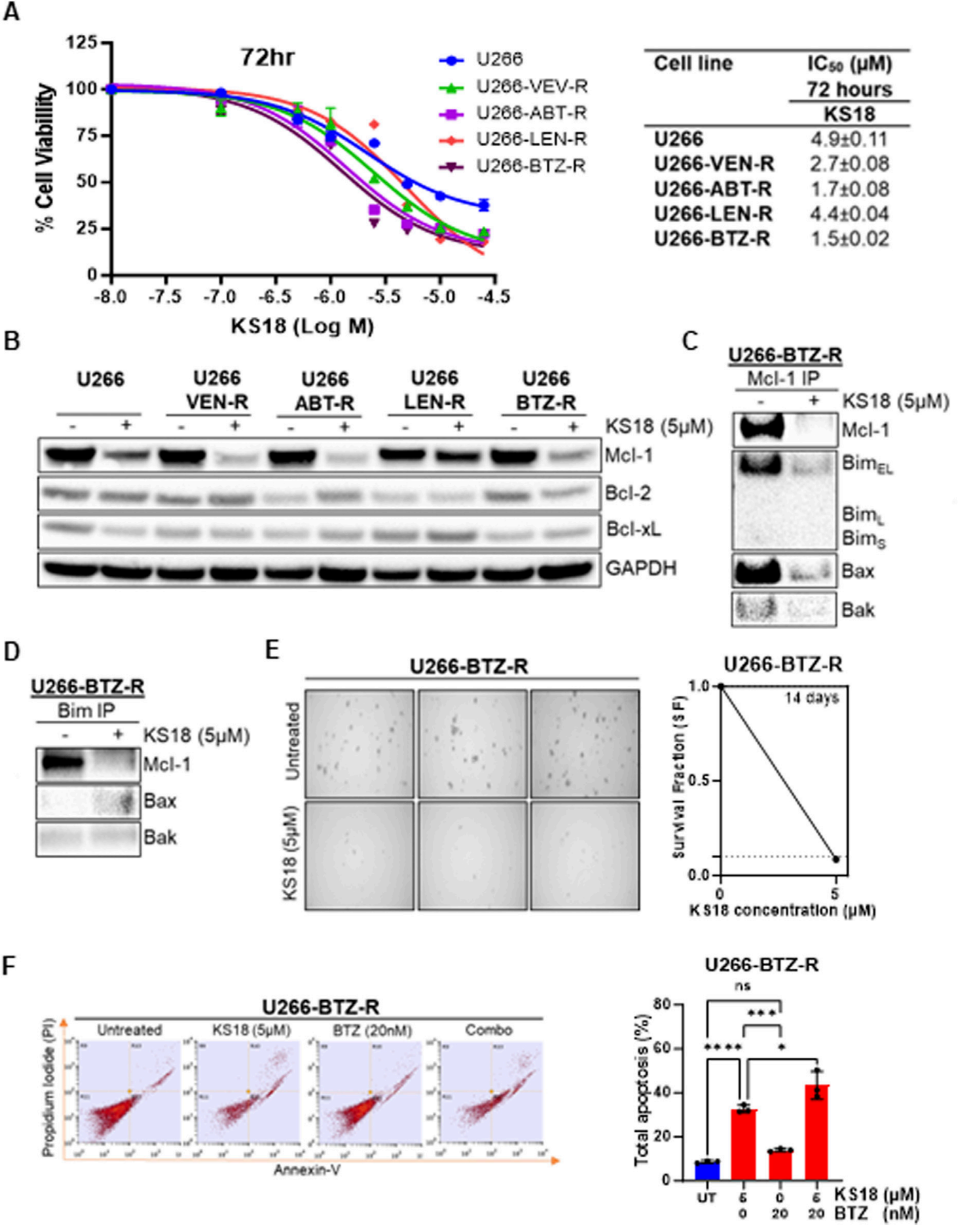
KS18 is effective against MM-resistant cells and re-sensitizes bortezomib resistant cells to bortezomib. **(A)**, A panel of human MM U266-resistant cell lines (U266-VTX-R, U266-ABT-R, U266-LEN-R, and U266-BTZ-R) were treated for 72 h with increasing dosages of KS18 (0–25 µM), and cytotoxicity was assessed using the MTT assay. **(B)**, KS18 (5 µM) was applied to several resistant cells for 24 h. **(C, D)**, Immunoprecipitation followed by western blotting was performed in U266 bortezomib resistant cells treated with KS18 (5 µM) for 24 h **(E)**, the colony forming ability of U266 bortezomib resistant (U266-BTZ-R) cells was assessed as described under Materials and Methods section. **(F)**, U266-BTZ-R cells were subjected to a 24-h treatment with BTZ (20 nM) or KS18 (5 µM), either individually or in combination, followed by staining with Annexin V dye and analysis using FACS. The total count of apoptotic cells was quantified (n = 3), and a one-way ANOVA was conducted utilizing GraphPad Prism software. In all experiments vehicle treated cells served as control. Wherever applicable GraphPad prism was used for graphical representation and IC_50_ calculation. *P ≤ 0.05, ***P ≤ 0.001, ****P ≤ 0.000, ns, not significant.

Resistance to standard therapies poses a significant challenge in the recurrence of MM, urging the exploration of alternative treatments. We investigated how effective KS18 is in MM-resistant cells. We generated resistant cells to a variety of MM chemotherapeutic drugs (bortezomib, lenalidomide, venetoclax, and ABT-737) and discovered that all resistant cell lines express a different pattern of anti-apoptotic Bcl-2 family proteins, notably high level of Mcl-1 (Supplementary Figures S5A, B). Moreover, the high expression of Mcl-1 compared to Bcl-2 or Bcl-xL suggests that Mcl-1 is a driver of resistance in these models. We assessed the efficacy of KS18 in resistant cells and determined that its remarkable effectiveness in these cells exceeds its impact on parental lines (Figure 5A). The IC_50_ value was notably lower in the resistant cells (1.5 ± 0.02 to 4.4 ± 0.02 µM) compared to the parent U266 cells (IC_50_: 4.9 ± 0.11 µM), suggesting increased sensitivity in the resistant cells, possibly attributable to KS18’s capacity to target Mcl-1. Moreover, KS18 reduced Mcl-1 expression in MM-resistant cells Figure 5B, this indicates the KS18’s potential as a viable therapeutic alternative for surmounting resistance in MM cells (Figure 5B). Furthermore, we investigated whether KS18 therapy may influence the dynamic interactions of Mcl-1 and pro-apoptotic proteins in resistant cells. KS18 therapy modified Mcl-1: Bim and Mcl-1: Bax interactions in resistant cells, and restored Bax apoptotic role (Figures 5C, D). We further evaluated the effect of KS18 on colony formation in U266-bortezomib-resistant (U266-BTZ-R) cells. Treatment with KS18 significantly inhibited colony formation in U266-BTZ-R cells, resulting in a 92% reduction compared to vehicle-treated controls. This substantial decrease in colony formation suggests that KS18 may play a critical role in delaying the development of resistance in these cells (Figure 5E). The combination of KS18 and bortezomib shows synergistic results in the treatment of bortezomib-resistant cells (Figure 5F). U266-BTZ-R cells were subjected to treatment either individually or in conjunction with KS18 (5 µM) or bortezomib (20 nM). Flow cytometry analysis utilizing Annexin V/PI staining reveals that the incorporation of KS18 reinstated the responsiveness of bortezomib in resistant cells, suggesting that KS18-based therapeutic combinations are efficacious against resistant cells and can re-sensitize multiple myeloma cells to chemotherapy (Figure 5F). Collectively, our findings endorse the application of KS18 in the treatment of MM-resistant cells, presenting a promising strategy to address resistance and enhance chemotherapeutic efficacy.

### KS18 presents a promising option as an additional treatment for bortezomib-resistant MM cells, enhancing the effectiveness of the apoptotic response to venetoclax

**FIGURE 6:**
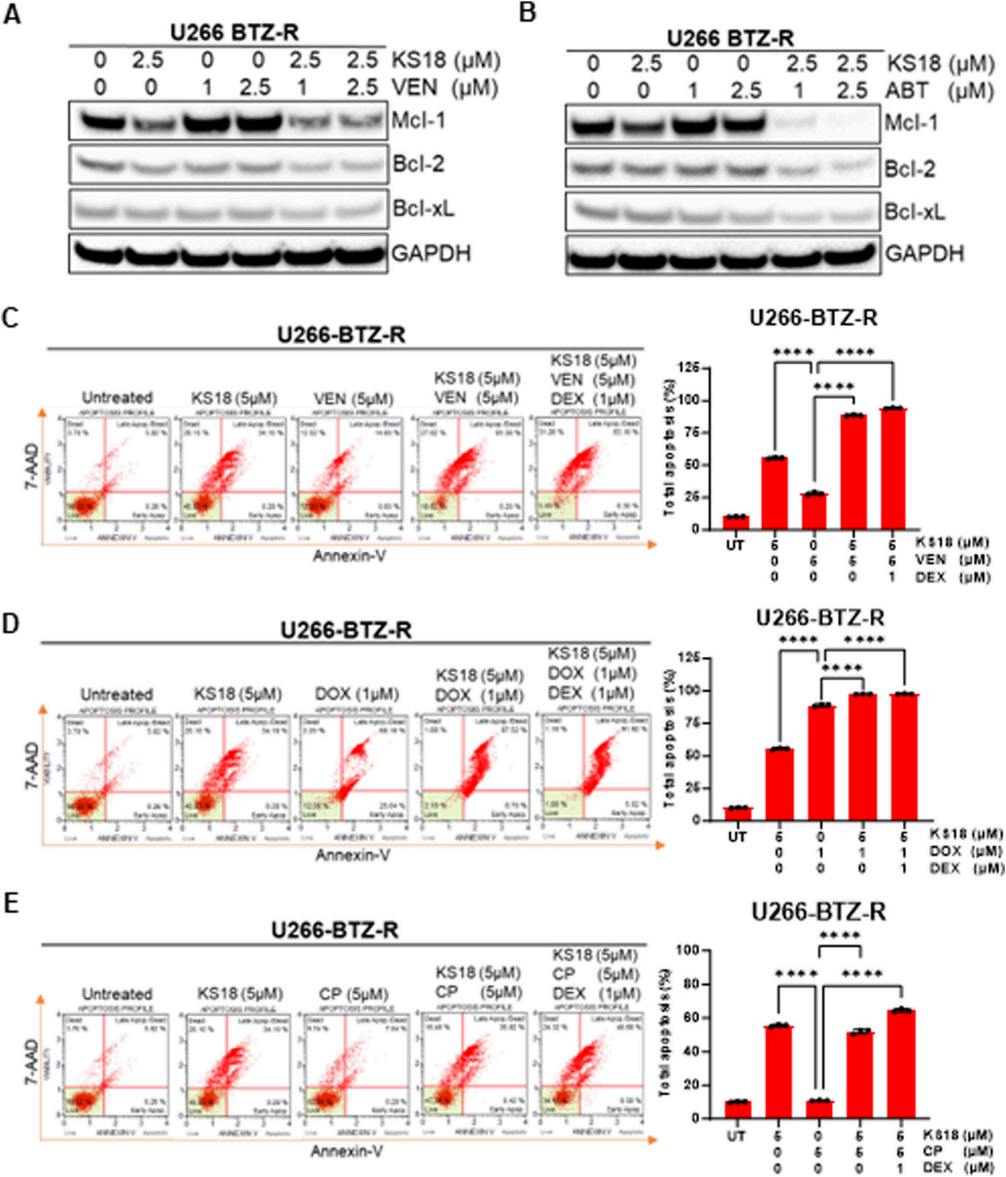
KS18 increases the efficacies of Bcl-2/Bcl-xL inhibitors in bortezomib resistant cells. **(A, B)**, U266-BTZ-R cells were subjected to treatment with venetoclax (VEN) at concentrations of 1 and 2.5 μM, or ABT-737 (ABT) at the same concentrations, both alone and in conjunction with KS18 (5 μM) for a duration of 24 h and immunoblotting was conducted to assess the specified anti-apoptotic proteins. **(C–E)**, U266-BTZ-R cells were subjected to a 24-h treatment with KS18 (5 μM) either alone or in conjunction with VEN (5 µM), cyclophosphamide (CP) (5 µM), doxorubicin (DOX) (1 µM), and dexamethasone (DEX) (1 µM), subsequently stained with Annexin V dye and evaluated using the Muse Cell Analyzer. The total count of apoptotic cells was quantified (n = 3), and one-way ANOVA was conducted, and graph was generated utilizing GraphPad Prism software. Vehicle treated cells served as control in all experiments. ****P < 0.0001.

Our prior research indicated that venetoclax elevates Mcl-1 expression in MM cells, potentially facilitating treatment resistance. Consequently, we aimed to evaluate the efficacy of KS18 in comparison to venetoclax or ABT-737 in resistant cells and to ascertain if the combination of KS18 with venetoclax/ABT-737 could surmount chemotherapy-induced resistance and enhance treatment outcomes. The efficacy of Bcl-2/Bcl-xL inhibitors was evaluated with KS18 in relation to three bortezomib-resistant cell lines. KS18 markedly decreased cell viability across all three cell lines. The IC_50_ values for KS18 (1.5–1.6 μM) were consistently lower than those for the Bcl-2/Bcl-xL inhibitor (1.7- >25 μM), demonstrating the superior potency of KS18 in overcoming bortezomib resistance (Supplementary Figure S5C). This suggests that among the resistant population, Mcl-1 inhibitors are a better option than Bcl-2/Bcl-xL inhibitors, particularly in Mcl-1 overexpressing phenotypes. Subsequently, we sought to evaluate the potential of combining KS18 with venetoclax in bortezomib-resistant cells. We administered several concentrations of venetoclax to U266-BTZ-R cells, both as a monotherapy and in conjunction with KS18 (2.5 µM). As anticipated, venetoclax elevated Mcl-1 levels; however, co-treatment with KS18 mitigated this increase, along with Bcl-2 and Bcl-xL (Figure 6A). Comparable results were noted with the combination of ABT-737 and KS18 in U266-BTZ-R cells (Figure 6B). Subsequent examination of KS18 as an additional therapy in venetoclax- and ABT-737-resistant cells revealed that the combination treatment markedly decreased the levels of Bcl-2, Bcl-xL, and Mcl-1 (Supplementary Figures S5D, E). The data suggests that KS18 possesses potential therapeutic efficacy in surmounting resistance to both venetoclax and ABT-737.

According to a recent study, dexamethasone enhances the expression of both Bcl-2 and Bim in MM, which shifts Bim binding towards Bcl-2 and promotes Bcl-2 dependency in MM (Matulis et al., 2016). We investigated the impact of dexamethasone on making MM bortezomib-resistant cells susceptible to venetoclax. We also evaluated whether adding KS18 to a dexamethasone and venetoclax regimen would increase apoptosis; indeed, adding KS18 to the regimen resulted in apoptosis in over 95% of U266-BTZ-R cells (Figure 6C). Vincristine, doxorubicin, and dexamethasone (Vad) is another combination used as induction therapy for newly diagnosed MM (Harousseau et al., 2010). We investigated the combination of KS18, doxorubicin, and dexamethasone in MM bortezomib-resistant cells. The KS18/doxorubicin/dexamethasone regimen demonstrated *in vitro* synergism in U266-BTZ-R (Figure 6D).

A three-drug regimen of bortezomib, cyclophosphamide, and dexamethasone (VCd or CyBorD) is an essential therapy option for patients with recurrent MM who are unresponsive to lenalidomide and daratumumab. Furthermore, VCd is an appropriate alternative for patients who are at higher risk of lenalidomide problems (i.e., acute kidney failure, increased thromboembolic risk) and for countries where lenalidomide is not allowed for initial therapy (Kumar et al., 2012). Our study underscores the synergistic effect of co-treatment with KS18, particularly evident in the KS18/cyclophosphamide/dexamethasone regimen, demonstrating *in vitro* synergism in U266-BTZ-R (Figure 6E).

In conclusion, these findings show that KS18 is an excellent Mcl-1 inhibitor, offering promise as an adjuvant therapy in combination with various agents including bortezomib, cyclophosphamide, doxorubicin, venetoclax against a spectrum of resistant MM cells.

### KS18 demonstrates potential as a therapeutic agent in MM xenografts

**FIGURE 7:**
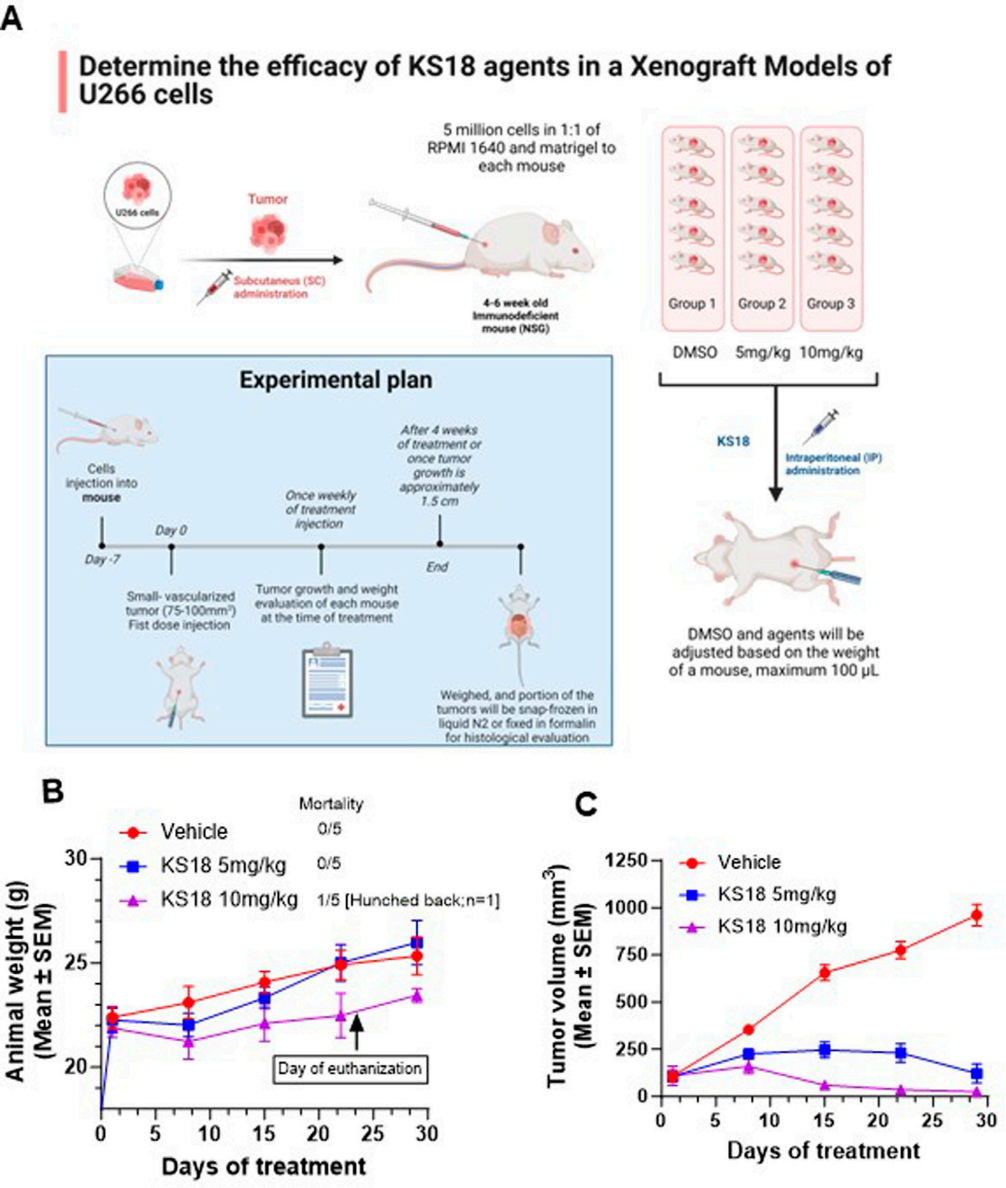
KS18 is safe and inhibits MM tumor growth. **(A)**, MM xenograft was developed as described in Materials and Methods section. Mice were treated with increasing dose of KS18 once a week for 4 weeks. **(B)**, KS18 dosages were delivered for 28 days in xenografted mice, with animal weights (g) recorded weekly. **(C)**, the graph depicts tumor volume (mm³) in mice treated with either vehicle or KS18. Mean ± SEM of animal body weight (g) and tumor volume (mm³) for vehicle (DMSO) and KS18-treated mice, presented using GraphPad Prism.

To assess the *in vivo* efficacy of KS18 in MM, we established a subcutaneous xenograft model by injecting 5 × 10^6^ U266 cells mixed 1:1 with matrigel into NSG mice. A power analysis was conducted to ascertain the suitable sample size. With a power of 0.8, an alpha level of 0.05, and an anticipated effect size of 50% tumor volume reduction accompanied by a standard deviation of 15%, a minimum of 5 mice per group is necessary to identify a statistically significant difference between the treatment and control groups. The mice were randomized into three groups after roughly 10 days post-tumor cell injection, when the tumor volume reached approximately 100 mm³ (n = 5 per group) (Figure 7A). Mice received KS18 via intraperitoneal injection at doses of 5 and 10 mg/kg once weekly for 4 weeks. The third group served as a control, administered with DMSO (n = 5 per group).

We assessed the safety and acceptability of KS18. A weekly dosage of 5 and 10 mg/kg was well tolerated by the mice (Figure 7B). Despite one mouse receiving 10 mg/kg exhibiting signs of distress (hunched posture) and being euthanized in the later stages of the trial (day 23), all mice generally retained normal weight relative to the vehicle-treated group (Figure 7B). KS18 markedly decreased tumor proliferation relative to the control group. Following 4 weeks of treatment, the mean tumor volume in the KS18 group decreased by 95% relative to the control group (*p* < 0.01; Figure 7C). Tumor volumes in the control group consistently escalated throughout the investigation, but tumors in the KS18 group exhibited delayed development and regression in all subjects. The data indicates that Mcl-1 inhibition may serve as a viable therapeutic strategy for treating MM, especially in addressing resistance to current treatments.

## Discussion

MM is challenging to treat. Recent advancements in treatment have not altered the fact that it remains an incurable disease; all patients ultimately develop resistance and experience recurrence (Kumar and Rajkumar, 2018; Abdi et al., 2013). Members of the Bcl-2 family of proteins are essential in mediating the apoptotic pathway. The overexpression of the Mcl-1 is significantly associated with unfavorable prognosis and resistance to treatment (Wuilleme-Toumi et al., 2005; Punnoose et al., 2016a; Punnoose et al., 2016b; Tahir et al., 2017). This study presents KS18, a new and effective Mcl-1 inhibitor, exhibiting considerable anti-tumor efficacy in both *in vitro* and *in vivo* models of MM. KS18 proficiently surmounts resistance to bortezomib and venetoclax, underscoring its prospective therapeutic applicability.

Our research offers new understanding of the molecular pathways that contribute to the anti-tumor effectiveness of KS18. The efficacy of KS18 significantly diminished upon Mcl-1 knockdown, thereby reinforcing that KS18’s anti-tumor activity is primarily mediated by Mcl-1 suppression. This outcome highlights the specificity of KS18 for Mcl-1 and strengthens its therapeutic promise in tumors that are critically reliant on Mcl-1 for survival, including MM and bortezomib-resistant malignancies. The diminished efficacy in Mcl-1-knockdown cells further indicates that KS18 is improbable to have considerable off-target effects, hence improving its safety profile. KS18 not only suppresses Mcl-1 expression but also interferes with essential regulatory mechanisms related to Mcl-1 stability and function. We specifically established that KS18 diminishes Mcl-1 expression by obstructing the interaction of the STAT3 transcription factor with the Mcl-1 promoter region. Considering STAT3’s function in promoting the transcription of pro-survival genes such as Mcl-1, this approach indicates that KS18 can proficiently interfere with a principal pathway that sustains elevated Mcl-1 levels in resistant cancer cells. Additionally, KS18 stimulates the phosphorylation of Mcl-1, facilitating its destruction through the ubiquitin-proteasome pathway. Phosphorylation is a recognized regulatory mechanism that designates Mcl-1 for ubiquitination, leading to its degradation and diminishing its anti-apoptotic function (Mojsa et al., 2014). The dual method of action - suppressing Mcl-1 expression and promoting its degradation - sets KS18 apart from other Mcl-1 inhibitors that operate mostly through a single mode of action (Kotschy et al., 2016; Al-Odat et al., 2021). By concurrently addressing transcriptional and post-translational regulation, KS18 exemplifies a holistic approach to diminish Mcl-1 levels in neoplastic cells. Nonetheless, it remains unclear how KS18 governs these two distinct processes, necessitating further investigation.

Our findings indicate that KS18 triggers apoptosis in MM cell lines via a sequence of pro-apoptotic mechanisms, encompassing the activation of Bim_EL_, Bax, the release of cytochrome c, and the subsequent activation of caspases resulting in PARP cleavage. The activation of Bim_EL_ and Bax by KS18 is significant, as Bim_EL_ is a recognized BH3-only protein that directly inhibits Mcl-1, enabling Bax to trigger mitochondrial outer membrane permeabilization (MOMP) (Czabotar et al., 2009; Kim et al., 2009). This subsequently induces the release of cytochrome c from mitochondria, resulting in the activation of caspases and the initiation of apoptosis. In comparison to other Mcl-1 inhibitors like S63845, AMG 176, and AZD5991, KS18 seems to stimulate analogous apoptotic pathways, however with notable differences (Yi et al., 2020; Li et al., 2019; Tron et al., 2018). S63845 causes apoptosis via Bim_EL_ and Bax activation; nevertheless, it necessitates constant dosage to sustain Mcl-1 suppression, potentially leading to problems associated with drug resistance and toxicity (Li et al., 2019). Conversely, KS18 induces fast activation of the apoptotic cascade, as seen by the prompt release of cytochrome c and significant caspase-3 activation, indicating that KS18 may possess a more effective method for initiating cell death with reduced dosing frequency.

Our research underscores the robust anti-myeloma efficacy of KS18, exhibiting more effectiveness than many recognized treatments, including venetoclax, ABT-737, melphalan, and pomalidomide. Moreover, KS18 initiated apoptotic pathways in primary patient samples, highlighting its potential clinical significance and wide application among diverse patient populations with varying disease characteristics. Venetoclax and ABT-737 have demonstrated limited effectiveness in MM, especially regarding Mcl-1-mediated resistance (Liu et al., 2022; Luedtke et al., 2017). Our findings suggest that KS18 demonstrates superior efficacy compared to these drugs, presumably owing to its capacity to selectively target Mcl-1. Melphalan and pomalidomide, two conventional treatments employed in MM treatment, exhibited diminished effects on cell viability relative to KS18. This discovery is noteworthy as it indicates that Mcl-1 inhibition could be a more effective approach for MM treatment, especially in instances when conventional chemotherapies or immunomodulatory agents do not yield lasting results. An intriguing feature of KS18’s mode of action is its capacity to synergize with bortezomib and dexamethasone, two fundamental components of MM therapy. Our combination tests demonstrated that KS18 markedly augmented the apoptotic effects of bortezomib and dexamethasone, resulting in a greater induction of apoptosis relative to monotherapy. This synergy is probably attributable to the complementary modes of action. KS18 efficiently reduces Mcl-1 levels, whereas bortezomib obstructs proteasome function, and dexamethasone alters glucocorticoid receptor signaling, thus enhancing apoptotic stress on myeloma cells. These data suggest the possible integration of KS18 into existing treatment protocols, which may enhance patient outcomes in therapy utilizing bortezomib and dexamethasone. KS18 distinguishes itself from other Mcl-1 inhibitors, such S63845 and AMG 176, by its capacity to synergize with both proteasome inhibitors and glucocorticoids. Although S63845 and AMG 176 have encouraging preclinical efficacy, their synergistic effects with conventional MM treatments have not been thoroughly validated.

Our research illustrates the significant effectiveness of KS18 against several drug-resistant MM cell lines. KS18 demonstrated significant efficacy in bortezomib-resistant cells, a difficult subtype of MM characterized by its need on Mcl-1 for survival. KS18 suppressed Mcl-1 expression in these resistant cells, resulting in the activation of pro-apoptotic proteins such as Bim_EL_ and Bax, which are essential for initiating the mitochondrial apoptotic pathway. Furthermore, KS18 markedly diminishes the colony-forming capacity of these resistant cells, specifically targeting clonogenic populations that facilitate relapse. KS18 synergistically interacts with bortezomib, augmenting its effectiveness in resistant cells by depleting Mcl-1 and sensitizing cells to proteasome inhibition. KS18’s dual mechanism of reducing colony formation and enhancing bortezomib efficacy underscores its potential to surmount resistance when conventional medicines are ineffective. These results endorse the continued advancement of KS18, especially in conjunction with proteasome inhibitors for the management of refractory MM.

Our findings indicate that KS18 significantly diminishes Mcl-1 expression triggered by venetoclax and ABT-737 in bortezomib-resistant MM cells, thereby addressing a critical resistance mechanism associated with Bcl-2/Bcl-xL inhibitors. Furthermore, KS18 improves the treatment efficacy when administered in conjunction with venetoclax and dexamethasone, doxorubicin and dexamethasone, as well as cyclophosphamide and dexamethasone. Conversely, other Mcl-1 inhibitors such S63845, AMG 176, and AZD5991 exhibit significant Mcl-1 inhibition; nevertheless, their capacity to synergize with a wide array of chemotherapeutic drugs remains inadequately explored. S63845 has demonstrated the ability to augment Bcl-2 inhibition; nevertheless, KS18’s extensive synergy with both chemotherapeutics and corticosteroids indicates a more adaptable function in the treatment of refractory MM.

The present study indicates that KS18 is markedly effective in inhibiting tumor growth in a MM xenograft model, exhibiting substantial tumor decrease relative to vehicle-treated mice. One mouse displayed toxicity after 23 days of treatment at a higher dose (10 mg/kg), whereas the lower dose of 5 mg/kg proved successful without further indications of toxicity, suggesting a beneficial therapeutic window. In comparison to other Mcl-1 inhibitors such as S63845, AMG 176, and AZD5991, which have demonstrated efficacy in preclinical models, toxicity continues to be a significant concern (Yi et al., 2020; Li et al., 2019; Tron et al., 2018). AMG 176 has been linked to dose-limiting toxicities in clinical trials (Chien et al., 2024), while KS18’s reduced effective dose seems to provide a superior safety profile. KS18 is positioned as a potentially safer and similarly effective Mcl-1 inhibitor, necessitating additional exploration in MM and associated cancers.

## Conclusions and future direction

This study revealed that KS18, a new Mcl-1 inhibitor, exhibited substantial activity in several MM cell lines, including those resistant to bortezomib and other treatments. KS18 significantly decreased Mcl-1 expression prompted by venetoclax and ABT-737, while simultaneously activating apoptotic pathways via Bim_EL_ and Bax, resulting in cytochrome c release, caspase activation, and PARP cleavage. It demonstrated enhanced efficacy relative to venetoclax, ABT-737, melphalan, and pomalidomide, and successfully suppressed colony formation in resistant cells. Furthermore, KS18 demonstrated synergy with bortezomib, dexamethasone, doxorubicin, cyclophosphamide, and venetoclax indicating its potential to augment current therapies and surmount drug resistance.

Nonetheless, caution is advised as our study encompassed a limited number of patient samples, which may not adequately reflect the variability of responses in a larger patient population. The possibility of off-target effects and the engagement of supplementary pathways must be meticulously assessed. Long-term effects and the safety profile of KS18, particularly in combination with other chemotherapeutic agents, need thorough investigation to ensure no adverse effects over extended periods. Although KS18 demonstrated encouraging outcomes in preclinical xenograft models by significantly inhibiting tumor growth at lower doses (5 mg/kg) without added toxicity, additional research is required to comprehensively assess its clinical efficacy and safety profile. Extensive clinical trials are required to resolve these issues, clarify any off-target effects, and validate the therapeutic efficacy of KS18 across a wider spectrum of patients and treatment scenarios.

## Data Availability

The original contributions presented in the study are included in the article/Supplementary Material, further inquiries can be directed to the corresponding author.
