# Supplementary material for: KS18, a Mcl-1 inhibitor, improves the effectiveness of bortezomib and overcomes resistance in refractory multiple myeloma by triggering intrinsic apoptosis
Source: Front Pharmacol. 2024 Oct 1;15:1436786. doi: 10.3389/fphar.2024.1436786 (PMC11473443; doi:10.3389/fphar.2024.1436786)
Supplement: Supplementary file 1 [file DataSheet1.zip › Supplementary Figure Legends.DOCX]

**Supplementary Figure Legends**

**Supplementary Figure 1: KS18 inhibits Mcl-1 and induces apoptosis in MM cells. *A.*** Superimposition of Mcl-1 (orange)- Noxa (magenta) in complex with KS18 (stick cyan) with Mcl-1. ***B.*** Myeloma cells show differential expression of anti-apoptotic Bcl-2 family protein. ***C,*** The effect of KS18 on Mcl-1 in two MM cell lines. ***D,*** The effect of KS18 on caspase-3 and PARP proteins in various MM cells. ***E&F,*** MM.1S cells were treated with KS18 (5µM) for 24 hours before being stained with caspases 3/7 dye or Annexin V dye as described in Materials and Methods sectionand evaluated using the Muse® Cell Analyzer. The total number of apoptotic cells was enumerated (n=3) and unpaired t test was performed using GraphPad prism software. **** P ≤ 0.0001. For sections ***B-D***, MM cells treated with or without KS18 (5µM) for 24 hours. Following incubation, cells were collected, lyzed, and immunoblotting was performed with mentioned antibodies. The vehicle treated cells served as control in all experiments.

**Supplementary Figure 2: KS18 increases the efficacy of bortezomib in MM cells. *A-C,*** Cell viability was evaluated using the MTT test after 72 hours of treatment with increasing dosages (0-25µM) of KS18 and other chemotherapeutic drugs (venetoclax, ABT-737, melphalan, and pomalidomide). ***D***, Bortezomib (BTZ) at concentrations of 5 and 10 nM, both alone and in conjunction with KS18 at 5 µM, was utilized to treat MM.1S cells, with cell viability assessed via the MTT assay. ***E***, MM.1S cells were treated for 24 hours with KS18 (5µM) alone or in combination with BTZ (20nM) and DEX (1µM), then stained with Annexin V dye and evaluated using the Muse® Cell Analyzer. The total number of apoptotic cells was enumerated (n=3). Vehicle treated cells served as control in all experiments. GraphPad prism was used to construct graphical representations and statistical analysis wherever needed. **** P ≤ 0.0001, ns: non-significant.

**Supplementary Figure 3: KS18 increases the efficacies of venetoclax in MM cells. *A&B,*** U266 and MM.1S cells were treated for 24 hours with VEN (0.5 and 1µM) alone and in conjunction with KS18 (5µM), and immunoblotting was performed against the specified antibodies. ***C-F,*** U266 and MM.1S cells were treated for 24 hours with VEN (0.5µM) alone or in conjunction with KS18 (5µM), then stained with caspases 3/7 dye or Annexin V dye and evaluated using the Muse® Cell Analyzer. The total number of apoptotic cells was enumerated (n=3). ***G,*** U266 and MM.1S cells were treated for 72 hours with increasing dosages (0-25µM) of VEN alone or in combination with KS18 (5µM), and cell viability was assessed using the MTT test. Vehicle treated cells served as control in all experiments. GraphPad prism software was used for statistical analysis and graphical presentations. **** P ≤ 0.0001.

**Supplementary Figure 4: KS18 increases the efficacies of ABT-737 in MM cells**. ***A&B,*** U266 and MM.1S cells were treated for 24 hours with ABT-737 (ABT) (1 and 2.5µM) alone and in conjunction with KS18 (5µM), and immunoblotting was performed against the specified antibodies. ***C-F,*** U266 and MM.1S cells were treated for 24 hours with ABT (1µM) alone or in conjunction with KS18 (5µM), then stained with caspases 3/7 dye or Annexin V dye and evaluated using the Muse® Cell Analyzer. The total number of apoptotic cells was enumerated (n=3). ***G,*** U266 and MM.1S cells were treated for 72 hours with increasing dosages (0-25µM) of ABT alone or in combination with KS18 (5µM), and cell viability was assessed using the MTT test. Vehicle treated cells served as control in all experiments. GraphPad prism software was used for statistical analysis and graphical presentations. **** P ≤ 0.0001.

**Supplementary Figure 5: KS18 outperformed venetoclax in MM-bortezomib resistant cells. *A&B,*** Mcl-1 and other anti-apoptotic proteins expression in MM-resistant cell lines determined by western blot. ***C,*** A panel of human MM bortezomib-resistant cell lines (MM.1S-BTZ-R, U266-BTZ-R, and RPMI8226-BTZ-R) were treated for 72 hours with increasing dosages (0-25µM) of KS18, VEN, and ABT, and cell viability was determined using the MTT assay. ***D,*** U266-VTX-R cells were treated with VEN (1 and 2.5μM) alone and in combination with KS18 (2.5μM) for 24 hours, and immunoblotting was performed. ***E,*** U266-ABT-R cells were treated with ABT (1 and 2.5μM) alone and in combination with KS18 (2.5μM) for 24 hours, and immunoblotting was performed. Vehicle treated cells served as control in all experiments. GraphPad prism was used to construct the graphical depiction and the IC_50_ calculation.
