# Supplementary material for: KS18, a Mcl-1 inhibitor, improves the effectiveness of bortezomib and overcomes resistance in refractory multiple myeloma by triggering intrinsic apoptosis
Source: Front Pharmacol. 2024 Oct 1;15:1436786. doi: 10.3389/fphar.2024.1436786 (PMC11473443; doi:10.3389/fphar.2024.1436786)
Supplement: Supplementary file 1 [file DataSheet1.zip › Supplementary Figures.PPTX]

## Slide 1
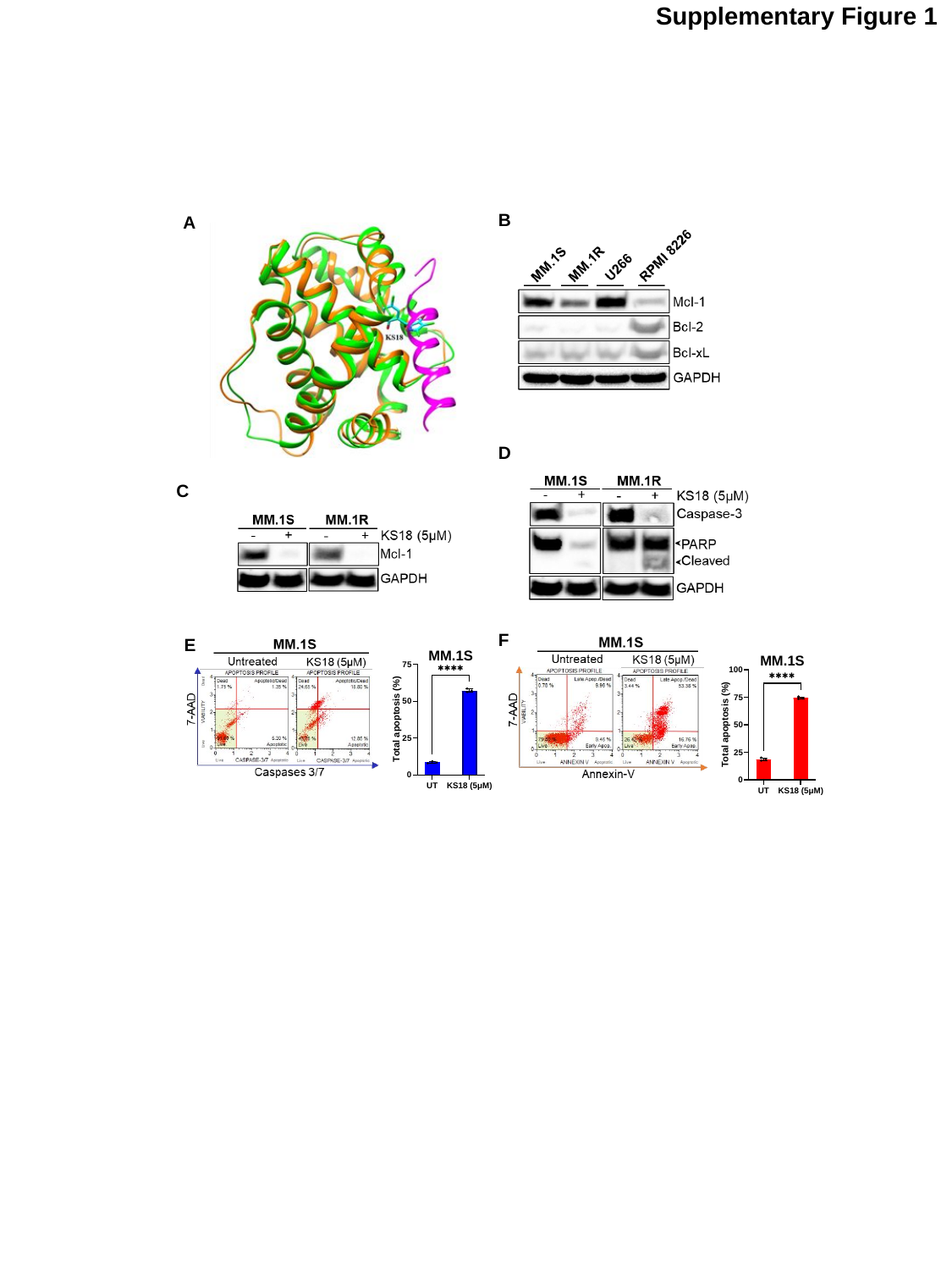

Supplementary Figure 1
B
A
D
C
F
E

## Slide 2
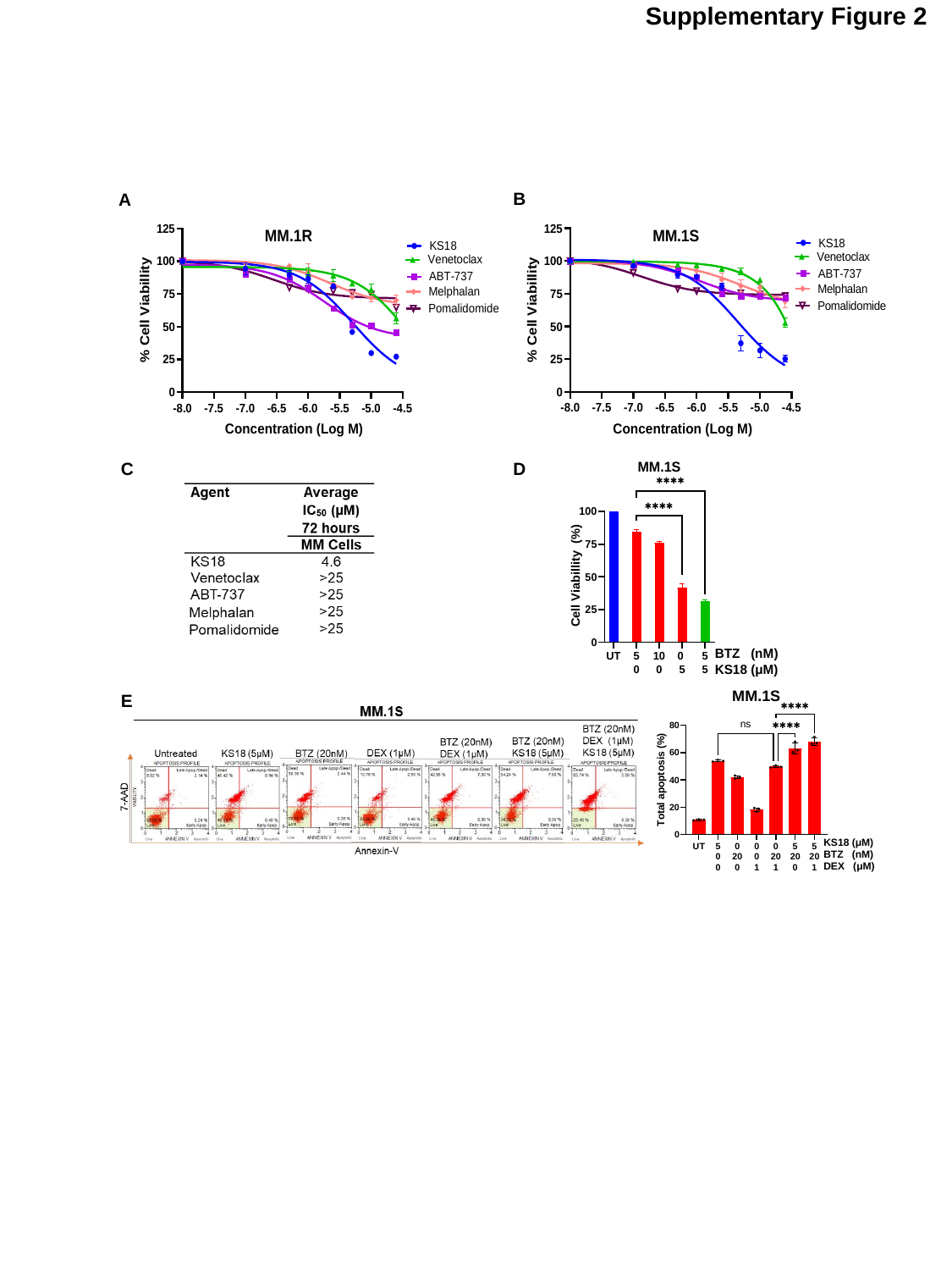

Supplementary Figure 2
B
A
C
D
E

## Slide 3
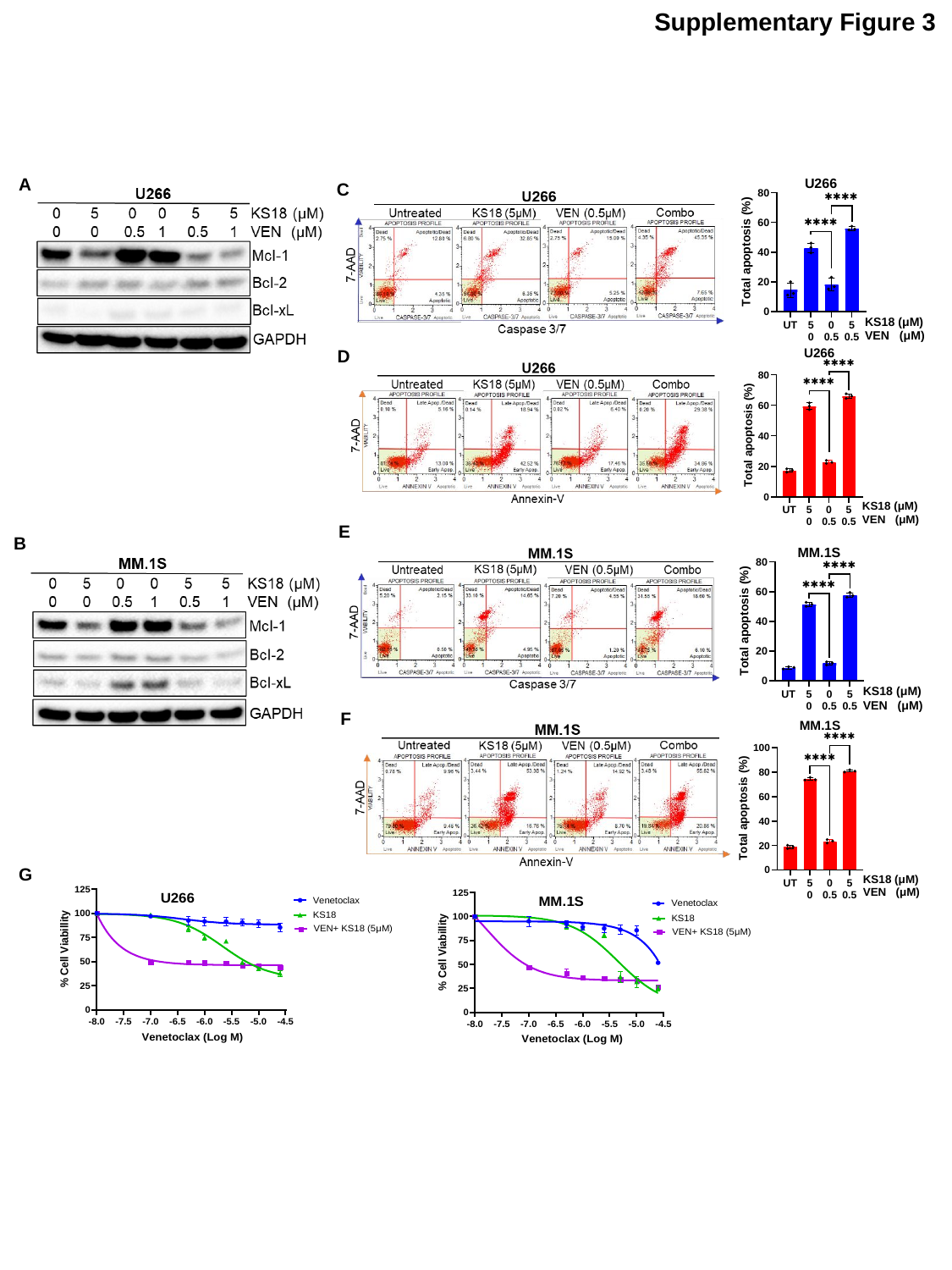

Supplementary Figure 3
A
C
D
E
B
F
G

## Slide 4
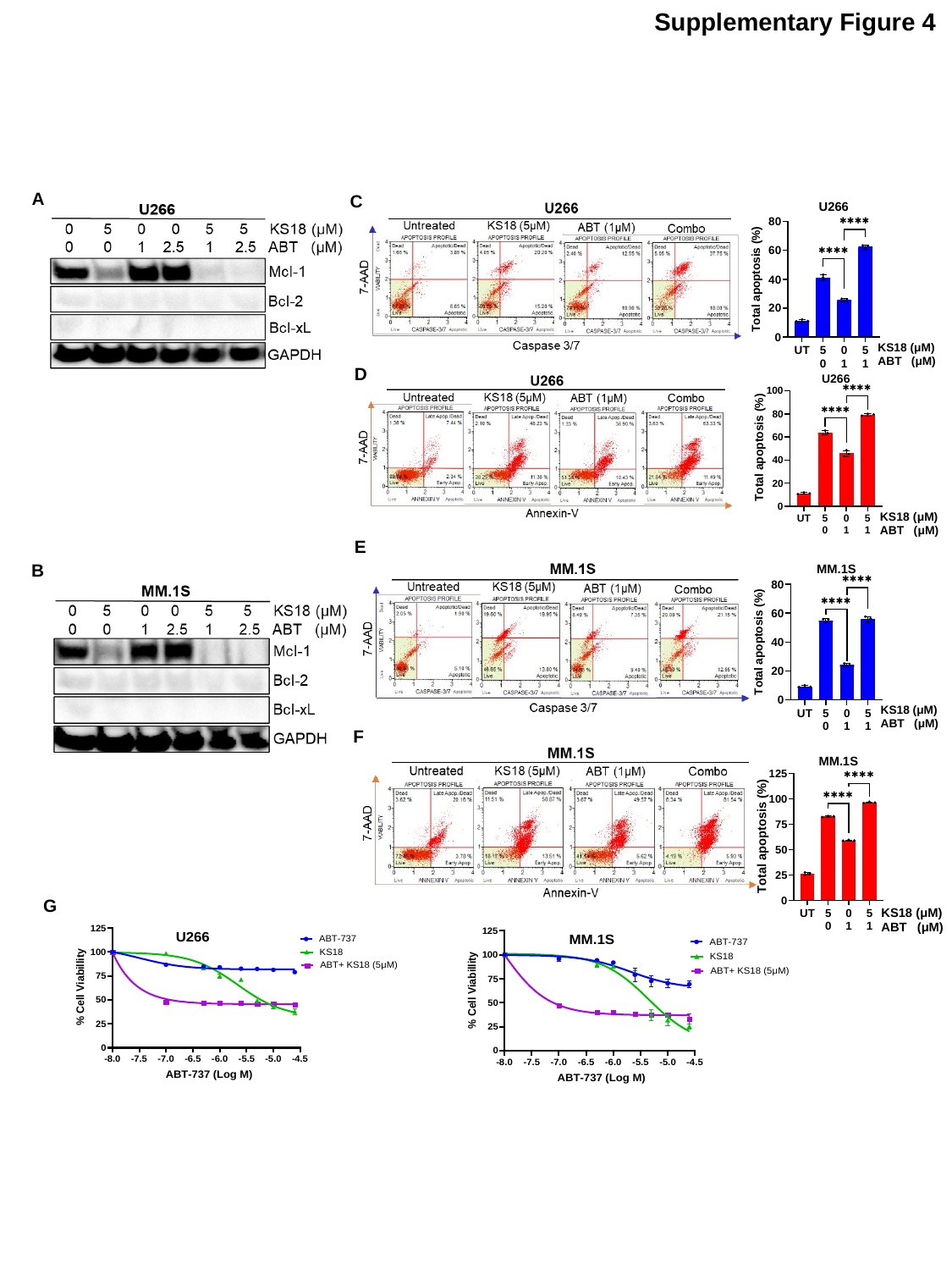

Supplementary Figure 4
A
C
D
E
B
F
G

## Slide 5
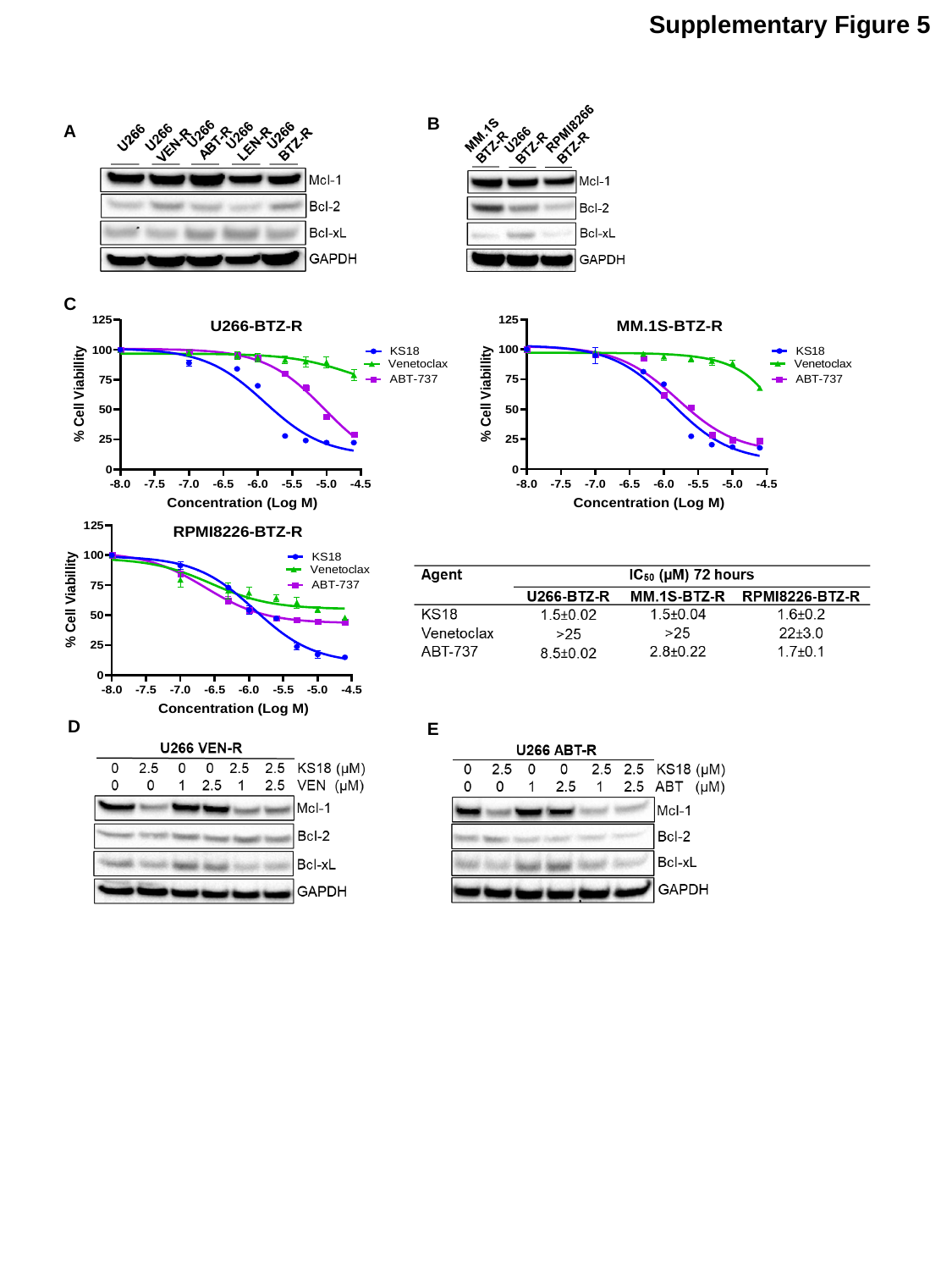

Supplementary Figure 5
B
A
C
D
E
